# Recombination hotspots in an extended human pseudoautosomal domain predicted from double-strand break maps and characterized by sperm-based crossover analysis

**DOI:** 10.1371/journal.pgen.1007680

**Published:** 2018-10-08

**Authors:** Nitikorn Poriswanish, Rita Neumann, Jon H. Wetton, John Wagstaff, Maarten H. D. Larmuseau, Mark A. Jobling, Celia A. May

**Affiliations:** 1 Department of Genetics & Genome Biology, University of Leicester, Leicester, United Kingdom; 2 Department of Forensic Medicine, Faculty of Medicine Siriraj Hospital, Mahidol University, Bangkok, Thailand; 3 Laboratory of Forensic Genetics and Molecular Archaeology, Department of Imaging and Pathology, KU Leuven, Belgium; Uniformed Services University of the Health Sciences, UNITED STATES

## Abstract

The human X and Y chromosomes are heteromorphic but share a region of homology at the tips of their short arms, pseudoautosomal region 1 (PAR1), that supports obligate crossover in male meiosis. Although the boundary between pseudoautosomal and sex-specific DNA has traditionally been regarded as conserved among primates, it was recently discovered that the boundary position varies among human males, due to a translocation of ~110 kb from the X to the Y chromosome that creates an extended PAR1 (ePAR). This event has occurred at least twice in human evolution. So far, only limited evidence has been presented to suggest this extension is recombinationally active. Here, we sought direct proof by examining thousands of gametes from each of two ePAR-carrying men, for two subregions chosen on the basis of previously published male X-chromosomal meiotic double-strand break (DSB) maps. Crossover activity comparable to that seen at autosomal hotspots was observed between the X and the ePAR borne on the Y chromosome both at a distal and a proximal site within the 110-kb extension. Other hallmarks of classic recombination hotspots included evidence of transmission distortion and GC-biased gene conversion. We observed good correspondence between the male DSB clusters and historical recombination activity of this region in the X chromosomes of females, as ascertained from linkage disequilibrium analysis; this suggests that this region is similarly primed for crossover in both male and female germlines, although sex-specific differences may also exist. Extensive resequencing and inference of ePAR haplotypes, placed in the framework of the Y phylogeny as ascertained by both Y microsatellites and single nucleotide polymorphisms, allowed us to estimate a minimum rate of crossover over the entire ePAR region of 6-fold greater than genome average, comparable with pedigree estimates of PAR1 activity generally. We conclude ePAR very likely contributes to the critical crossover function of PAR1.

## Author summary

95% of our genome is contained in 22 pairs of chromosomes shared by all humans. However, women and men differ in their sex chromosomes: while women have two X chromosomes, men have an X and a smaller, sex-determining Y chromosome. To ensure correct partition of X and Y into sperm, genetic exchange (crossover) must occur between these very different chromosomes in a short, shared region. The location of the boundary of this region was thought to have been conserved since before the divergence from old world monkeys at least 27 million years ago, but recently it has been shown that some human males carry an extended version on their Y chromosomes, thanks to the transposition of a piece of DNA from the X chromosome. Here, we asked if genetic exchange occurs in this newly extended region. To do this, we used previously published information that signposted the positions within the X chromosome segment which exhibit the hallmarks of crossover initiation. We then sought direct evidence of crossover in the sperm of men carrying the extension. This work showed that the signposts were accurate, pointing to frequent crossover in this novel shared sex-chromosomal domain.

## Introduction

The major pseudoautosomal region (PAR1), located at the tips of the short arms of the human sex chromosomes, is a region of interchromosomal homology [1, 2]. In contrast to its smaller counterpart (PAR2) on the long arms of the sex chromosomes [3], PAR1 plays an essential role in male meiosis by supporting pairing and obligatory exchange between the X and Y [4], failure of which can lead to sex-chromosomal aneuploidy such as Klinefelter syndrome (47,XXY), and is associated with increased infertility [5–7]. The human PAR1 is ~2.7 Mb in length, and until recently it was thought to have been stable during most of primate evolution [8]. Indeed, since its initial molecular characterization, it was widely accepted that the boundary was fixed approximately at its present location before the divergence of the old world monkeys and great apes 27–32 million years ago [9] and is delineated by an *Alu* element insertion on the human Y chromosome. However, despite this, there is evidence that its boundary, PAB1, has shifted distally in the past, as the proximal 240 bp of sex-specific DNA shows 77% sequence similarity between the human X and Y [8,10]. More recently, direct evidence of pseudoautosomal region plasticity came from a chance discovery during an aCGH (array comparative genomic hybridization) screen for copy number variation (CNV) in ~4,300 patients with developmental disorders, which showed that a small subset of men carry an extended PAR1 (ePAR): this demonstrates that the PAR1 boundary is not static, but polymorphic in modern humans [11].

**Fig 1:**
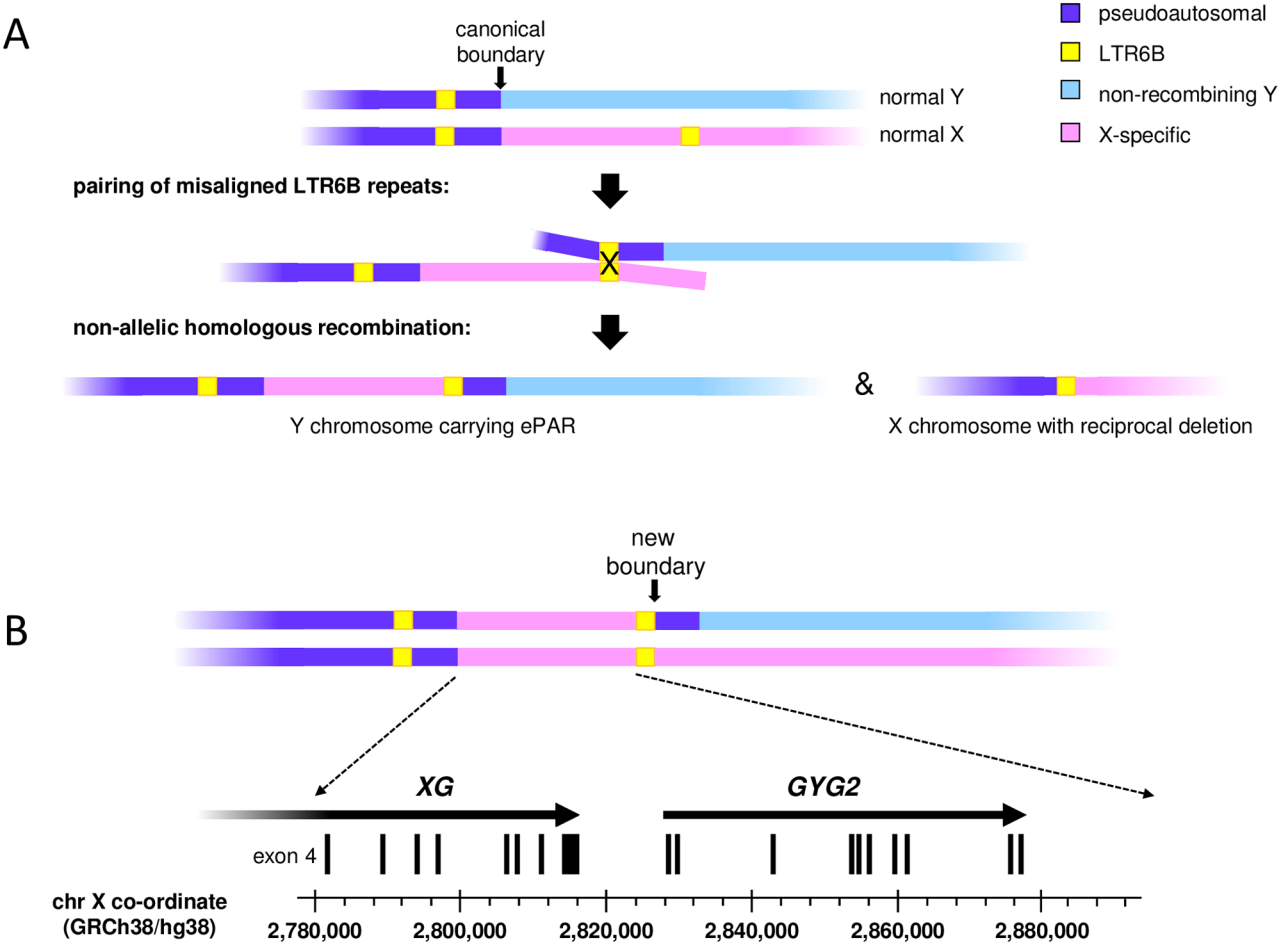
Schematic representation of formation and organization of the ePAR. (A) Normal pairing of the short arms of the X and Y chromosomes in male meiosis is limited to PAR1 (purple) such that homologous recombination can occur up to the canonical boundary as marked. However, mispairing of the PAR1 LTR6B element (yellow box) carried on a Y chromosome (blue) with one located more proximally on an X chromosome (pink) can result in non-allelic homologous recombination and the generation of gametes containing either an ePAR-carrying Y chromosome, or the reciprocal deleted X chromosome. Schematics not to scale. (B) Pairing and therefore homologous exchange between an ePAR Y chromosome and a normal X chromosome is predicted to extend proximally to a new boundary. The first three exons of the *XG* blood group gene fall within PAR1, while the remaining exons are carried on the X chromosome; men carrying the ePAR thus have two full-length XG genes like females. Similarly, whilst most men are hemizygous for the *GYG2* gene, ePAR carriers have two copies of this gene.

The Mensah *et al*. study [11] established that creation of the ePAR involved transfer of ~110 kb of X-chromosomal PAR1-proximal sequence and concomitant duplication of a ~5-kb portion of PAR1 to the Y chromosome. Furthermore, this insertional translocation was deemed most likely to be the result of non-allelic homologous recombination (NAHR) mediated by flanking ~550-bp LTR6B elements, and consistent with this, a family segregating the predicted reciprocal ~115-kb deleted form of the X was also identified [11] (Fig 1A). In contrast to most males but akin to females, men carrying the ePAR have two full-length copies of the apparently clinically irrelevant *XG* blood group gene [12], as well as two copies of the *GYG2* gene, encoding a precursor for glycogen synthase particularly important in the liver [13,14] (Fig 1B). Interestingly, both these genes escape inactivation in females [15]. In the Mensah *et al*. study, the ePAR was observed in 15 independent Belgian and French families: all Belgian ePAR Y chromosomes belonged to one sub-haplogroup, I2a (I-P37.2), while those of the two first-degree relative French carriers belonged to a sub-haplogroup within the distantly-related lineage R1b, namely R-P312 [11]. This indicated that the creation of ePAR is recurrent and has occurred at least twice based on the global Y-chromosomal phylogeny [16].

Mensah *et al*. presented indirect evidence that the translocated region within the ePAR actually functions pseudoautosomally. PacBio sequencing of <5% of the total ePAR indicated that at least two haplotypes exist amongst the haplogroup-I2a men; these were interpreted as a consequence of recombination between X and ePAR rather than mutation accumulation, because they differed by twelve single nucleotide polymorphism (SNP) variants all of which are also observed on X chromosomes [11]. More recently a gradual decline in X-chromosome genetic diversity spanning the canonical boundary was noted [17]: this contrasts with the expected abrupt drop at the boundary given the lower effective population size of strictly X-linked sequences (*i*.*e*. two copies in females but only one in males) compared with a truly pseudoautosomal sequence (two copies in both sexes) and provides further evidence consistent with the ePAR supporting exchange between the X and Y.

Here we build on these initial studies to seek direct evidence that the ePAR supports meiotic exchange by identifying *de novo* sperm recombinants (crossovers [COs], and noncrossovers [NCOs]) that map to this region from two men carrying ePAR-bearing Y chromosomes belonging to the I2a haplogroup. Since double-strand breaks (DSBs) induced by the protein SPO11 are known to initiate meiotic recombination [18], we target two subregions of the X chromosome involved in the translocation that are known, via single-stranded DNA sequencing (SSDS) data, to support DSBs in the male germline of presumed non-ePAR-carrying individuals [19]. Furthermore, we sequence >90% of the entire translocated region to extend our understanding of the recombinational history of the region as a whole.

## Results

### Identification of sperm donors for analysis of recombination in ePAR

The ePAR has been found in two Y-chromosome haplogroups, I2a and R1b, that are frequent in Europe [20, 21] so we focused on North European semen donors in our collection. Man 20 had previously been found to have a duplication of at least 17 kb of X-chromosome sequence that spanned PAB1 and was therefore a candidate ePAR-carrier (S1 Fig). A second candidate, man 53, was identified on the basis of a similar Y microsatellite or Short Tandem Repeat (Y-STR) haplotype and therefore predicted to share Y-chromosome haplogroup I2a-L233 with man 20. ePAR status was confirmed by sequencing of the proximal insertion junction; both sperm donors carry the “Junc1” sequence shared by eight of the nine independently sampled haplogroup I2a ePARs studied by Mensah *et al*. [11].

### Targeting subregions of ePAR for de novo recombination analysis

**Fig 2:**
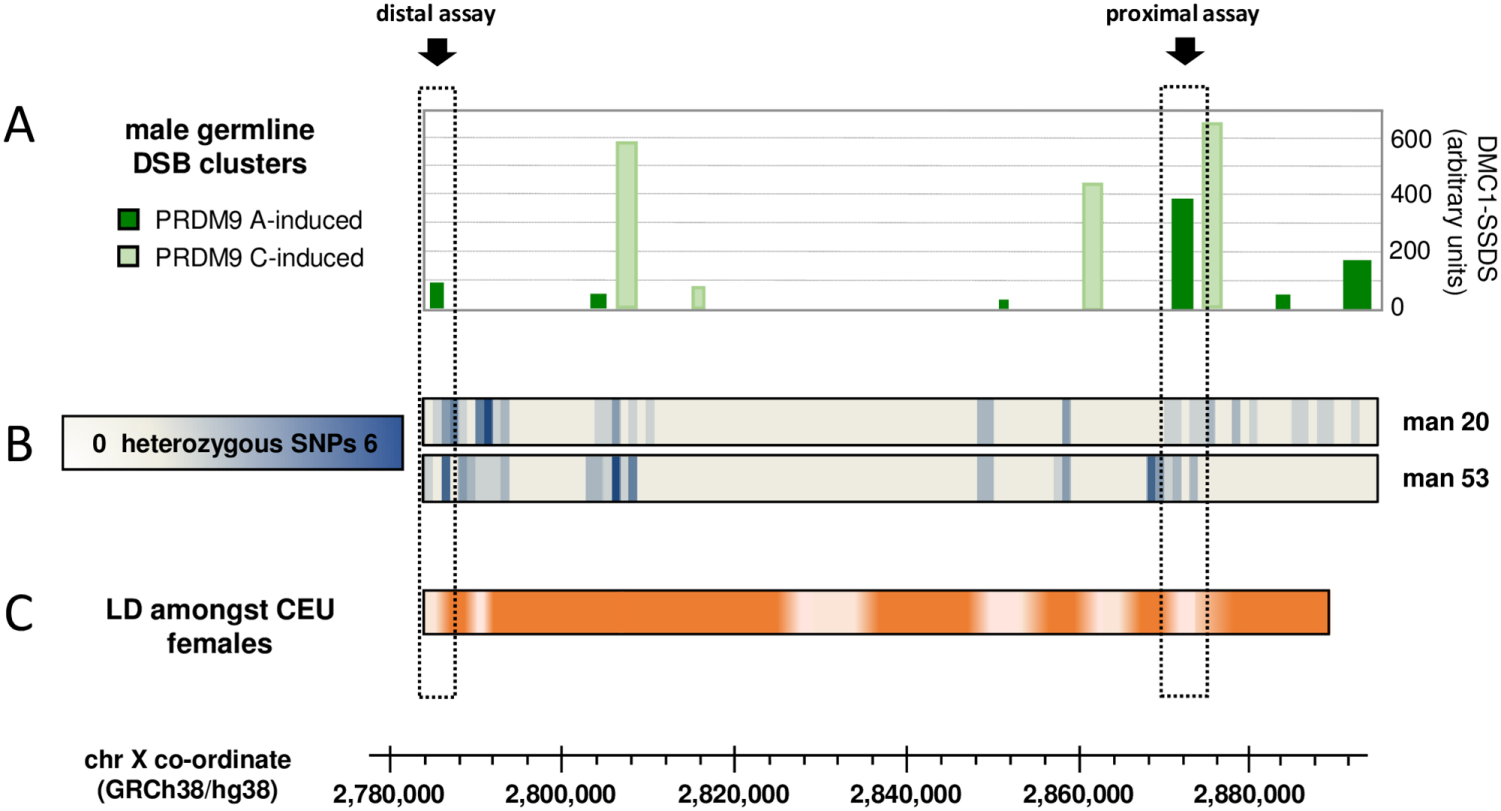
Choosing target regions within ePAR to assay for male germline *de novo* recombinants. (A) Distribution and intensity of DSB clusters that fall within the X-derived portion of the ePAR. Clusters were defined [19] by anti-DMC1 SSDS using testis biopsies from five men and designated as being induced by PRDM9 A (dark green) or PRDM9 C (light green); DSB strength is shown as the mean across relevant individuals using the arbitrary values reported in the original work [19]. (B) Frequency of heterozygous SNP markers per 1-kb interval identified in each of the two ePAR-positive sperm donors (man 20, man 53) as determined by Ion Torrent sequencing. (C) Linkage disequilibrium (LD) heat map derived from the 50 CEU females from the 1000 Genomes Project [34] (more intense orange equates to stronger LD). Data are based on |D´| values determined from SNPs with minor allele frequency >0.2 that passed tests for Hardy-Weinberg equilibrium specifically derived for markers on the X chromosome [72]; these stringent criteria meant that LD corresponding to the most proximal portion of the ePAR interval could not be examined. Scaling is shown with respect to GRCh38/hg38, and the two chosen assay intervals, distal and proximal, are indicated by the dashed boxes and arrows. See also S2 Fig.

Since human recombination events cluster into narrow 1–2 kb-wide hotspots [22–27] we sought to identify potential hotspot sites within the ePAR. Hotspot location is largely determined by PRDM9 [28–30], a presumed chromatin-remodelling protein, which binds DNA via its highly polymorphic zinc finger domain, and thus targets the induction of DSBs to specific locations (for a review see [31]). As both ePAR-carrying sperm donors were known to be homozygous for the common A-type zinc finger allele at the *PRDM9* locus, we considered the distribution and strength of meiotic DSB clusters induced by the PRDM9 A allele on the X chromosome region that makes up the ePAR, as ascertained by read depth in a previous SSDS study using testis biopsy material from presumed normal-PAR1-carrying men [19] (Fig 2A). We also considered the distribution of so-called hotspot motifs believed to be the cognate binding sites for the most common form of PRDM9 [30], as well as the SNP density across the entire region as reported in dbSNP [32], because recombination and DNA diversity often show a positive correlation [33]; neither showed a clear correspondence to the SSDS signals (S2 Fig). Finally, since our ability to detect recombinants is wholly reliant on informative SNPs in the sperm donors under study, we determined the distribution of heterozygous SNPs for >90% of the entire ePAR in both man 20 and man 53 using Ion Torrent sequencing (Fig 2B). These data suggested that recombination assays could be developed for both donors in each of two DSB clusters, as indicated in the figure. The distal assay region was located ~ 2.6 kb proximal to the canonical X-specific PAB1 and coincided with a moderately strong PRDM9-A-induced DSB cluster. The proximal assay region was some 86 kb upstream of this and coincided with the strongest DSB cluster as determined by SSDS read depth.

We also compared the male meiotic DSB data with the pattern of historical female-dominated X-chromosomal recombination activity as determined by linkage disequilibrium (LD), using SNP data from the 1000 Genomes Project [34] (Fig 2C). Both the distal and proximal assay intervals coincide with regions of LD breakdown, suggesting that these intervals have been active in the female germline too. In fact, five of the six regions of LD breakdown were found to correspond to either PRDM9-A- or PRDM9-C-induced DSB clusters (PRDM9 C and related alleles are known to activate different subsets of hotspots compared with the A allele, and collectively encode the next most common class of PRDM9 protein [35]). Conversely, only six of the ten male DSB clusters coincide with historical recombination activity in the female germline. We found no clear relationship between DSB strength and LD breakdown; of the six PRDM9-A-induced DSB clusters, the two weakest map to regions of historical recombination in the female germline, but the next two weakest do not.

### Sperm crossovers in the distal region cluster into a classical hotspot

Each sperm donor was found to be heterozygous for at least two SNPs both upstream and downstream of the DSB cluster in the distal region. This allowed development of a full CO assay for each, whereby forward allele-specific primers (ASPs) from one parental haplotype are used in conjunction with reverse ASPs from the opposite haplotype to selectively amplify *de novo* recombinants from multiple PCR reactions each containing several hundred molecules [36]. This is an efficient means by which to both estimate CO frequencies and to recover CO molecules for breakpoint mapping by subsequent typing of intervening SNPs.

**Fig 3:**
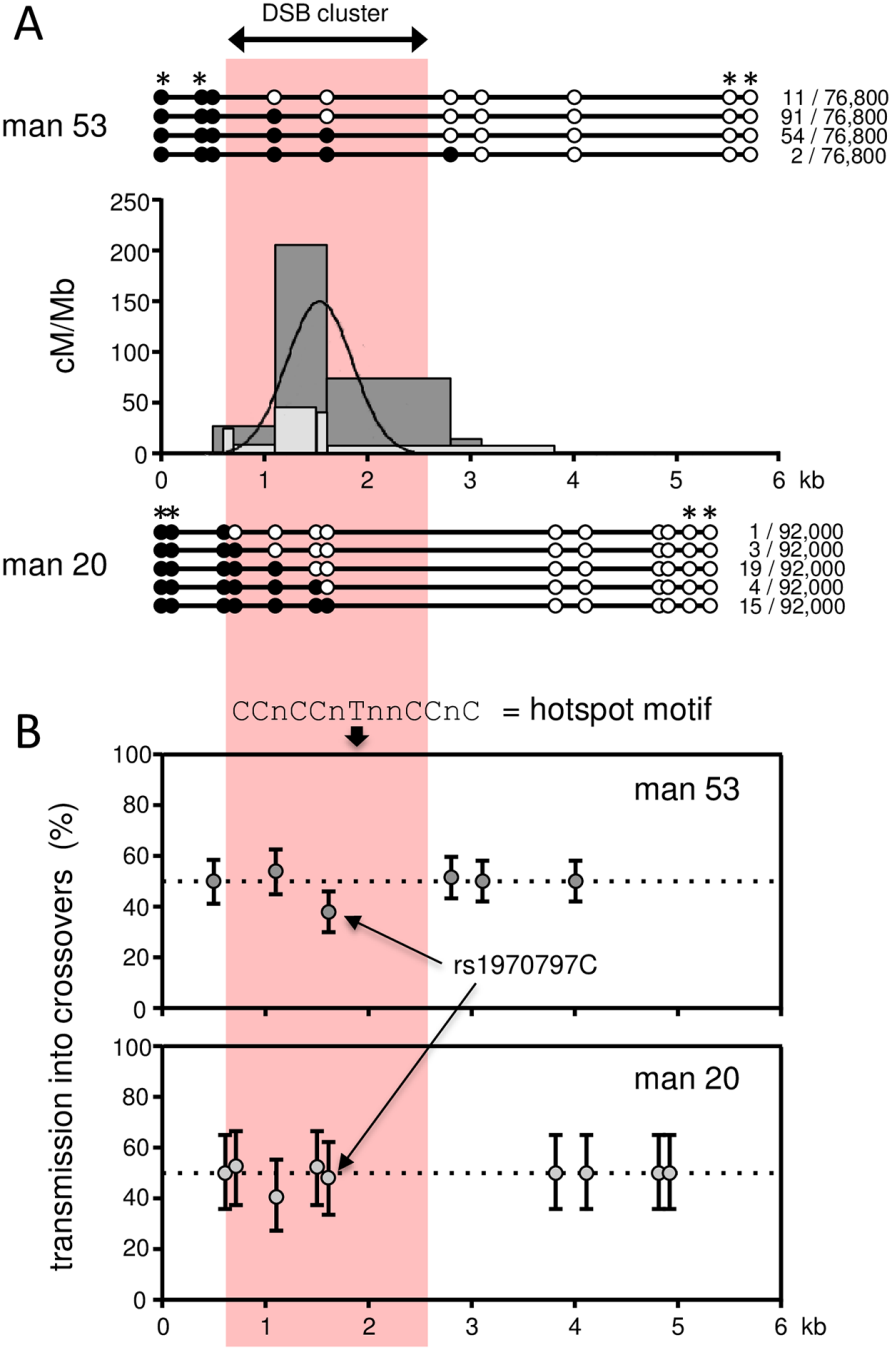
*De novo* sperm crossover activity in the distal region. (A) Sperm CO profiles for each of the two ePAR-carrying men analysed. A total of 158 recombinants were recovered from 76,800 sperm from man 53 using reciprocal crossover assays (*i*.*e*. Ab plus Ba COs, where AB and ab are the parental haplotypes) compared with 42 from a total of 92,000 sperm from man 20. Recombination activity expressed in cM/Mb along the assayed intervals is shown in the central graphs with the crossover activity of man 53 shown by the dark grey histogram and that of man 20 by the light grey histogram. The combined least-squares best-fit normal distribution for both men is shown by the black curve. The recovered CO structures together with their frequencies are shown above (man 53) and below (man 20) with heterozygous SNP locations represented by circles. SNPs marked with an asterisk were exploited for recombinant recovery (see Methods and S6 Table). The pink panel spans the interval in which male meiotic DSBs were previously mapped by DMC1-SSDS [19] and coincides with the peak CO activity determined from *de novo* sperm events in this study. (B) Transmission frequencies of SNP alleles into reciprocal COs with 95% credible intervals determined by Bayesian analysis. Transmission of the ‘strong’ allele (C or G) is shown for transition polymorphisms and transmission of the purine allele (A or G) is shown for the transversion polymorphisms. The upper panel shows the transmission data from man 53, the lower panel those for man 20. All markers with the exception of rs1970797 in man 53 were consistent with the expected 50:50 transmission of the two alleles into reciprocal events. This polymorphism lies 126 bp proximal to the predicted hotspot centre and 210 bp distal of the closest hotspot motif [73], as indicated at the top of the upper panel. CO asymmetry has previously been noted at hotspots that do not contain obvious matches to this motif, yet are nonetheless specifically activated by PRDM9 A [38,42]. Note that our failure to observe asymmetry for man 20 may simply be a consequence of the small number of COs detected for this donor.

Reciprocal assays were carried out for each of the two men. Collectively, 200 *de novo* COs were isolated and mapped from a total of 168,800 sperm molecules screened. Ninety-five percent of events clustered into a 1.3-kb-wide interval, entirely consistent with both autosomal and pseudoautosomal sperm CO hotspots [22,23,37], and with the peak of CO activity almost exactly mapping to the centre of the DSB cluster (Fig 3A). Despite a shared distribution of events (the inferred centre points of each donor’s distribution are estimated to be offset by <10 bp), the two men exhibited a ~4-fold difference in rate (man 53 RF = 0.21% (95% CI 0.18–0.24%), man 20 RF = 0.05% (95% CI 0.03–0.06%), P << 0.0001, 2-tailed goodness of fit test). This is within the observed range noted at other characterized sperm CO hotspots, controlling for both *PRDM9* status and *cis*-effects influencing initiation (see below) [38], and is comfortably within the 30-fold range of DSB strength as measured by SSDS read depth across the five men tested over this interval [19].

Ordinarily, reciprocal events should show a 50:50 ratio of alleles at heterozygous SNP sites; however, several CO hotspots have been shown to exhibit significant transmission distortion (TD) between alleles for markers close to the hotspot centre [37,39–42]. This phenomenon is most readily explained by differences in the frequency of recombination-initiating DSBs between the two parental haplotypes, since the repair of such lesions uses the intact homologue which in turn leads to over-transmission of the recombination-suppressing haplotype. TD is also referred to as CO asymmetry because the centre point of events is shifted between the reciprocal orientations even though the rates remain the same. Man 53 showed evidence of TD at the rs1970797 C/T polymorphism, with significant over-transmission of the T-allele (0.62 *cf*. 0.50, *P* = 0.008, two-tailed exact binomial test) and a displacement of the centre points of the reciprocal distributions of 126 bp. This SNP is the closest informative marker to the overall hotspot centre (Fig 3B). Given the CO rate estimate for man 53 and this level of TD observed amongst his COs, this equates to a gametic ratio of 50.024:49.976 and demonstrates that this hotspot, like some autosomal hotspots [39,40], is subject to a form of meiotic drive that will ultimately lead to its demise [43].

### Detection of sperm crossover and non-crossover events in the proximal region

The distribution of informative markers for both men in the proximal interval was such that similar CO assays could not be developed without requiring >20 kb amplicons that would at best result in very low PCR efficiencies. Instead, we designed assays in which ASPs are used in conjunction with universal primers to amplify one haplotype, and recombinants are detected by the presence of alleles from the non-amplified haplotype [36]. Since the latter is dependent on hybridization, this approach is less efficient as pool sizes are of the order of tens, not hundreds, of sperm per PCR, but it offers the advantage that both CO and NCO events can be detected.

**Fig 4:**
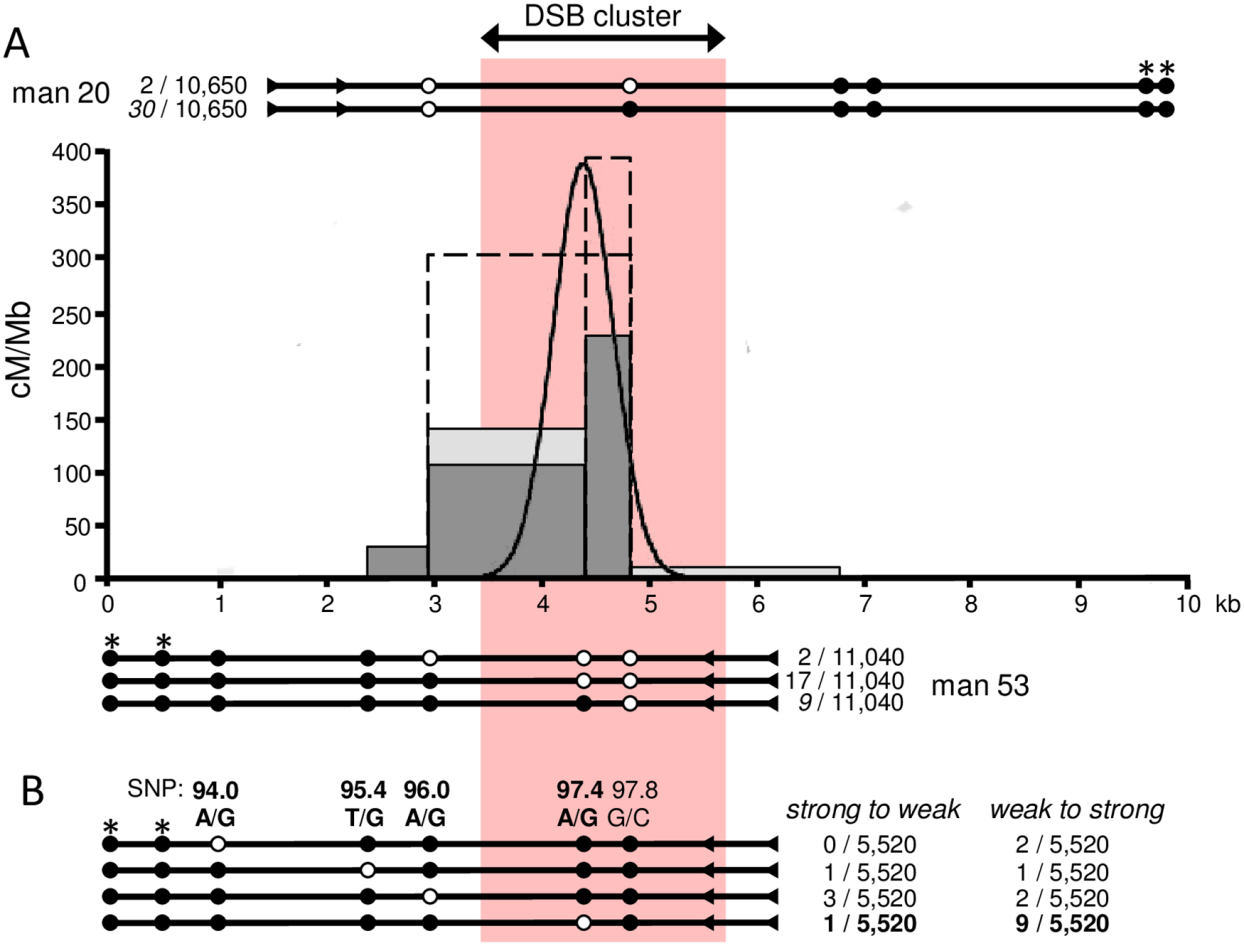
*De novo* recombination in the proximal region. (A) Sperm CO profiles in relation to the proximal DSB cluster as determined by DMC1-SSDS [19] (pink panel) for man 20 (light grey histogram) and man 53 (dark grey histogram), with the combined least-squares best-fit normal distribution shown by the black curve. As for Fig 3, data from reciprocal assays have been pooled and the recovered structures and their frequencies for each man are shown above and below the histograms with informative SNPs represented by circles. In these assays, ASPs were designed against the SNPs marked with asterisks and were used in conjunction with universal primers (triangles) to selectively amplify each parental haplotype; recombinants were then detected by probing for the alleles of the opposite haplotype represented here by white circles (see Methods, S6 and S7 Tables). Note that CO events involving only the terminal marker closest to the universal primers are indistinguishable from NCO events in this assay, so we arbitrarily designated half of such events as COs in these cases (numbers given in italics) but indicate with dashed boxes in the graph how the profiles would appear should all such events actually be COs. In the latter case, the hotspot width would be reduced by 250 bp, the centre point would be shifted proximally by 116 bp, and the peak activity would be ~ 830 cM/Mb. (B) Testing for GC bias amongst NCOs. Of the four informative SNPs for man 53 that carry a ‘weak’ and ‘strong’ allele, SNP 97.4 shows over-transmission into NCOs of the ‘strong’ allele G relative to the ‘weak’ allele A (*P* = 0.011, one-tailed binomial exact test). This SNP lies 97 bp proximal to the hotspot centre as shown by the black curve in (A). Whilst we cannot be sure of the number of NCOs involving the terminal marker SNP 97.8, both alleles at this SNP base pair with three hydrogen bonds (*i*.*e*. are ‘strong’) and there is no evidence of disparity between the orientations assuming at least half the terminal recombinants are NCOs (*i*.*e*. 9). For man 20, terminal marker SNP 96.0 recombinants were recovered in the two orientations with similar frequencies, again suggesting an absence of TD. Note a further two NCOs each affecting a different single site (SNP 99.8 or SNP 100.1) were also recovered for this man but are not depicted in this figure.

Across the two men, a total (*i*.*e*. CO+NCO) of 120 recombinants were detected from 21,690 sperm, and comparable recombination fractions were noted for each (man 20, 0.60% (95% CI 0.47–0.77%) and man 53, 0.51% (95% CI 0.39–0.66%), *P* >0.05, 2-tailed goodness of fit test). Despite the need to design different assays (Fig 4), in both cases, the most common type of event involved a switch of haplotype only at the terminal marker adjacent to the universal primer. In such cases it is impossible to distinguish COs from NCOs; furthermore, from analysis of other recombination hotspots, both are expected to co-localise, albeit with varying proportions [24,37,44,45]. In order to gain insight into the hotspot morphology, we therefore arbitrarily assigned half of such events as COs. Under this scenario, the proximal DSB cluster encompasses a 1.1-kb-wide hotspot with a peak activity of ~385 cM/Mb (see Fig 4A).

Sperm recombination data from the two assay intervals show comparable trends to those observed by Pratto *et al*. for the two DSB clusters. However measured, the proximal region shows more modest variability in recombination (at most a 1.2-fold difference between the sperm donors), compared with the distal region where a 4-fold difference in CO was noted, whilst DSB strength differed ~7- and ~30-fold respectively amongst the four men analysed by Pratto *et al*. [19]. Similarly, overall higher rates of recombination are observed in the proximal than distal region, though at best there is only ~12-fold difference compared with ~50-fold noted in mean DSB strength. Of course, only DSBs repaired using the homologue can be identified in our assays, and NCO events that do not encompass informative SNPs will go undetected but still contribute to the single-stranded DNA signal used to generate the DSB maps.

Unsurprisingly, given the distribution of markers, all twenty-one events that could be scored unambiguously as NCOs encompassed just a single polymorphic site with maximal conversion tract lengths ranging from 1853–2812 bp. Nineteen of these were observed for man 53 with peak numbers seen at SNP 97.4, the marker that lies nearest to the predicted hotspot centre (Fig 4B), entirely consistent with previous characterization of human meiotic NCOs [37,44,45]. Indeed, the closest adjacent marker to SNP 97.4 lies just 413 bp away, yet no co-conversions were observed suggesting, as seen in other studies, that most of the NCO tracts not only occur at the centre of the CO hotspot but are in fact short [44].

Assays were carried out in both orientations so it was possible to also test for TD in the proximal region. In contrast to the distal assay, none was observed amongst the COs for either man; however, significant bias was observed amongst the NCOs for the central-most SNP, 97.4, for man 53. Nine of the ten NCOs spanning SNP 97.4 contained the G- rather than A-allele indicating a preferential repair of ‘weak’ to ‘strong’ base pairs (Fig 4B) as noted in other studies [46]. TD confined to NCOs has previously been noted at two autosomal hotspots indicating differences in CO and NCO heteroduplex formation and/or mismatch repair; it is noteworthy that in both of these cases there was also a significant GC bias [45].

### Inferring past recombination events throughout ePAR

To gain a comprehensive understanding of the recombination history of ePAR we set out to sequence the entire region for the two sperm donors, six of the originally reported families of the Mensah *et al*. study [11], plus a further three carriers including one who is part of a CEPH pedigree (see Methods). Including family members to aid with subsequent phasing of alleles, this equated to twenty individuals, and ten independent I2a ePARs plus one R1b ePAR (S1 Table). We designed overlapping amplicons spanning the 110-kb transferred region of the X and sequenced them on an Ion Torrent platform to a mean read depth of 300x. We observed some unintended amplification from the long arm of the male-specific region of the Y (see Methods); this technical issue reflects the fact that the region of the X chromosome that transferred to form the ePAR shares a common evolutionary origin with this proximal portion of Yq, dating back ~30 Mya [47]. We therefore excluded approximately 9 kb from further analysis and determined SNP haplotypes for the remaining ~92% of the ePAR using the program PHASE [48,49]. We made use of family relationships where appropriate to determine which haplotype most likely corresponded to the ePAR. Two of the ePAR men for whom there were no first-degree relatives to analyse shared the same uncommon British surname indicative of shared ancestry (~1000 carriers in Great Britain in the year 1998) [50]. Whilst genealogical records suggest a putative common ancestor more than five generations ago, Y-STR profiling provides evidence of close paternal line relatedness of these two men (S4 Table); we took this into account when assigning their ePAR haplotypes.

**Fig 5:**
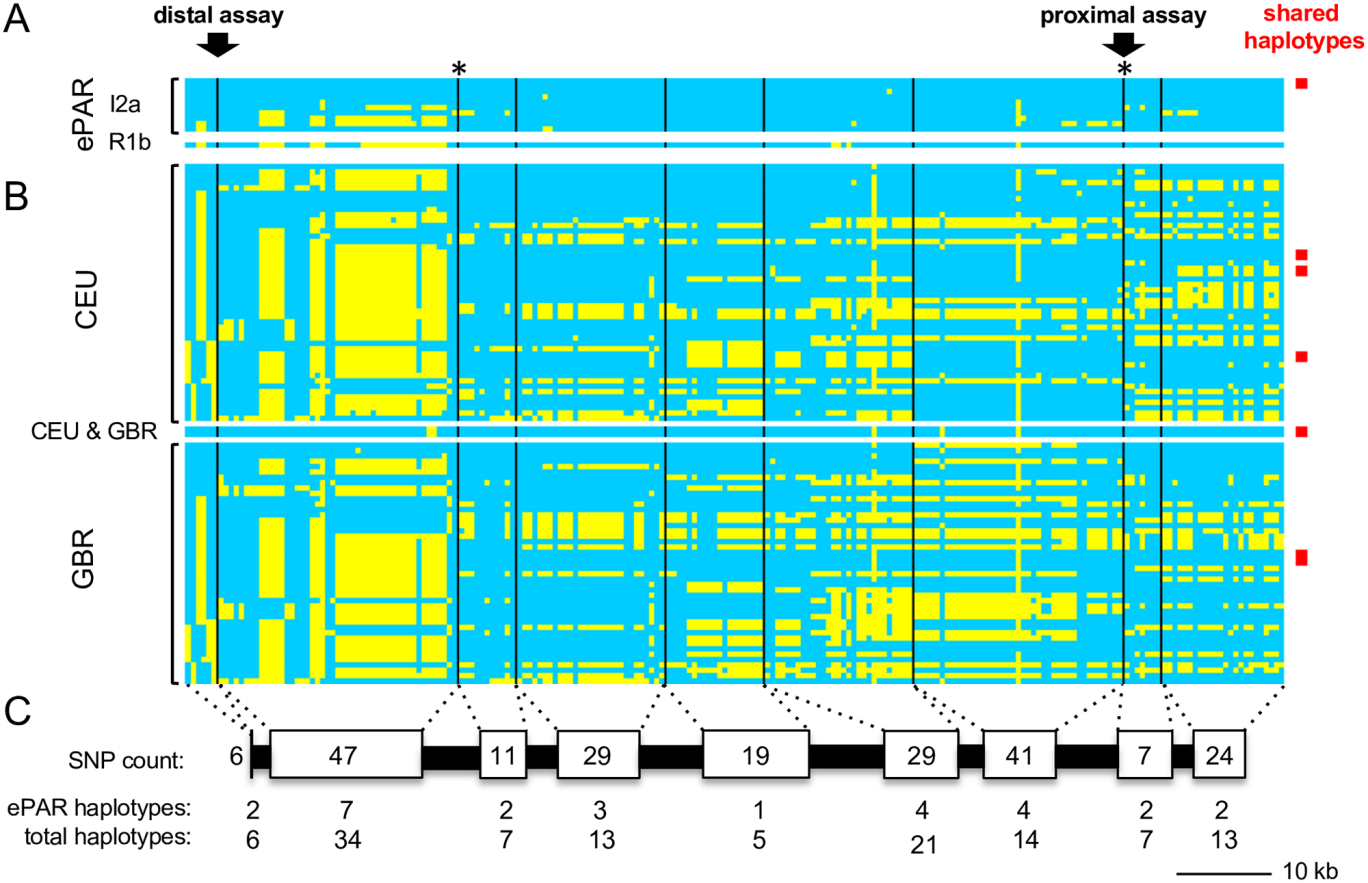
Comparison of inferred ePAR haplotypes with phase-known haplotypes from the corresponding region of the X chromosome. (A) SNP haplotypes from each of the eleven independently sampled ePARs (ten from the haplogroup I2a Y chromosome lineage and one from the R1b Y lineage) are shown in rows, clustered according to the distal haplotype block. Individual 6889_01 is shown at the top with all his alleles colour-coded blue; yellow denotes the alternative SNP alleles not carried by this man. Black vertical lines correspond to the relative locations of mapped PRDM9 A and C DSB clusters; in two instances, marked by asterisks, an A and C cluster lie in very close proximity. Arrows indicate the distal and proximal sperm recombination assay regions and the red box indicates a second ePAR that is identical to that of 6889_01. In total, nine of the ten ePAR haplotypes are unique to this dataset. (B) Phased X haplotypes from the 1000 Genomes Project [34] for the 49 CEU males and 46 GBR males. One haplotype is shared between the two sample sets as indicated. In addition, three pairs of identical X haplotypes were noted among the CEU and one X haplotype was found to be carried by three different GBR men (red boxes). In total, 42 of the 46 CEU haplotypes and 43 of the 44 GBR haplotypes are unique. None of these X haplotypes matches any of the ePAR haplotypes. (C) Relative scaling of the regions depicted together with summary count of the number of SNPs, number of ePAR haplotypes and the corresponding total number of haplotypes seen amongst the ePAR, CEU and GBR datasets per block of SNPs.

**Fig 6:**
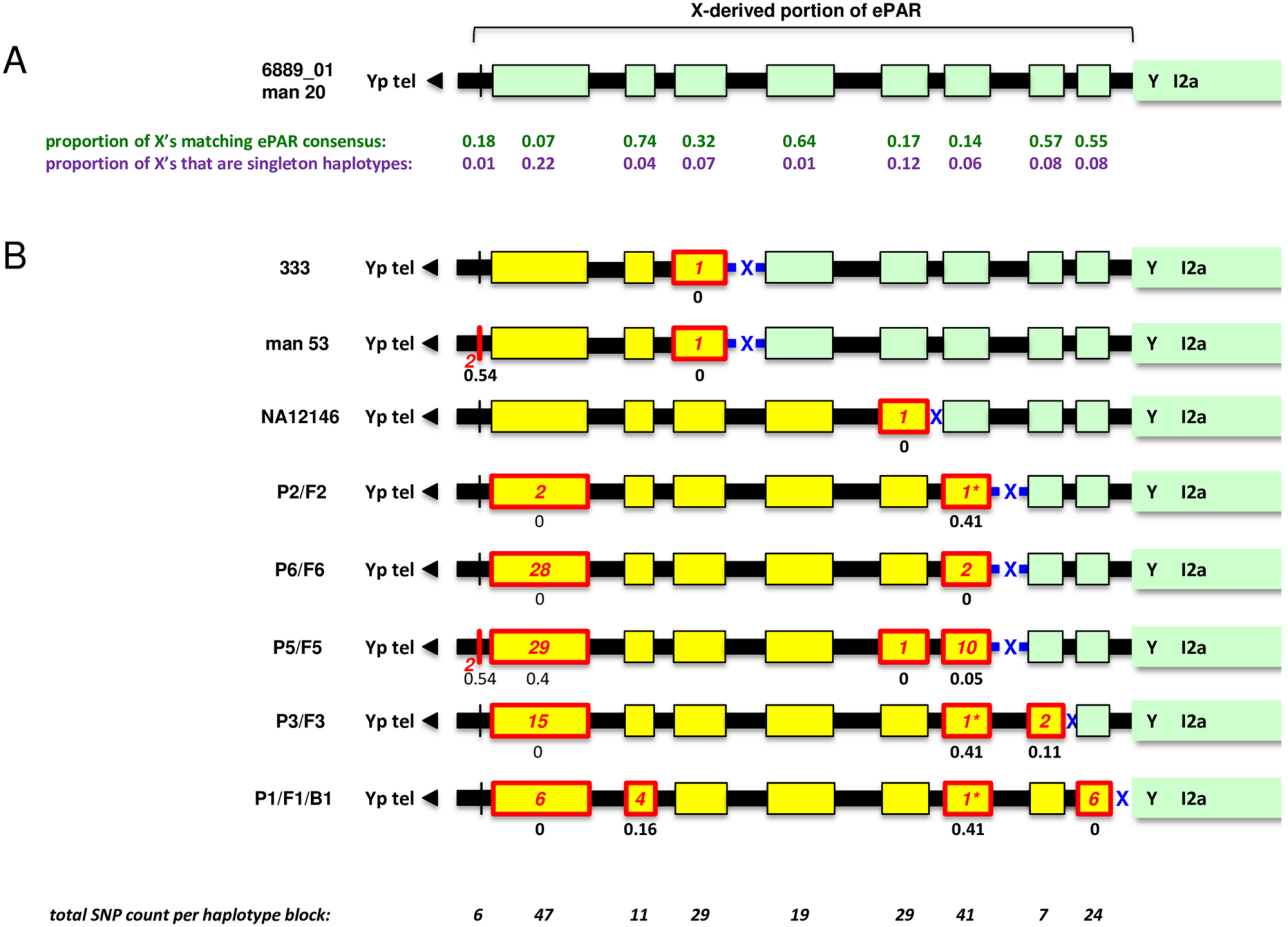
Simple interpretation of the I2a ePARs. (A) Schematic of consensus X-derived portion of ePAR carried by individuals 6889_01 and man 20. Green boxes with black outlines represent the shared haplotypes at each of the nine blocks of SNP markers whilst the intervening black boxes coincide with mapped PRDM9 A and C DSB clusters [19]; widths of all boxes are proportional to their length. The black triangle to the left points towards the canonical 2.7-Mb PAR1 and ultimately to the Yp telomere; the start of Y-specific I2a sequence is shown to the right. The frequency of phase-known X haplotypes among the 95 CEU+GBR males that match the modal ePAR haplotype for each block of SNPs are shown in green; the frequencies of singleton haplotypes amongst the same are shown in purple. (B) The remaining eight I2a ePARs, assuming they are the result of a single crossover between the consensus and an incoming X-linked haplotype depicted by yellow boxes (crossover interval shown with blue cross). Boxes with red outlines show haplotype blocks that differ from the consensus with the number of SNP changes shown in red; asterisks identify three haplotype blocks that differ from the consensus by the same single base pair change. Black numbers beneath boxes indicate the observed frequency of the non-consensus haplotype amongst the 95 phase-known X haplotypes from the CEU+GBR males. Total SNP counts per block are shown in italics at the bottom.

We focused on SNPs that overlap with those in the 1000 Genomes Project dataset for CEU (Utah Residents [CEPH] with Northern and Western European Ancestry) and GBR (British in England and Scotland), reasoning that Western European X chromosomes were most relevant for understanding the history of ePARs identified in the same geographical region (Fig 5). To aid interpretation, we focussed on SNPs that fall outside of the DSB clusters, as the signature of CO is most easily detected by new combinations of pre-existing and well-defined flanking LD haplotype blocks. This left a core set of 213 markers (S2 Table) split across nine regions or “blocks”, ranging in size from 558 to 16,143 bp. Of the ten independently sampled I2a ePARs, only two were found to have the same compound haplotype which was designated as the consensus (Fig 6A). The eight remaining I2a ePARs differed by up to four of the nine blocks (mode and median = 2) with changes from the consensus ranging from 1 to 29 SNP sites per block (mode = 1, median = 2). No complete matches were observed with phase-known X chromosomes from either the CEU or GBR males, though matches at the level of individual blocks were observed (9/19 that differ from the consensus, Fig 6B, S3 Table).

The simplest explanation of the diversity of the I2a ePARs would be that each unique haplotype is the outcome of a single, different CO event with an X chromosome (Fig 6). We therefore looked for matches for the predicted incoming (i.e. strictly X-linked) haplotype among the 95 phase-known CEU/GBR male X-haplotypes, but failed to identify any. Only five different compound haplotypes were seen more than once amongst this data set, consistent with high diversity in this region [17] and so it is possible that single exchanges involving unsampled X chromosomes could account for our observed ePAR haplotypes. Using published mutation rates for 23 Y-STRs we estimated the time to most recent common ancestor (TMRCA) for the I2a ePARs of our ten sequenced lineages at 3,877 ± 779 yrs, (S4 Table) [51–53], equating to 125 generations averaging 31 years. Assuming a minimum of eight recombination events to account for the nine extant I2a ePAR variants amongst the ten lineages examined, we thus obtain a minimum recombination rate of 0.64% (i.e. 8/(125 x10)).

This recombination rate is likely an underestimate of the true rate for two reasons; not all ten sequenced lineages radiated in one generation directly from the common ancestor (S3 Fig), and we have no way of definitively identifying multiple recombination events in these data. Interestingly, the two most diverged I2a sub-haplogroups also carry the most differentiated ePARs and importantly the variation from the consensus extends close to the proximal boundary, so it is entirely possible that these ePARs have experienced additional distal recombination events. Conversely, although the 23-Y-STR haplotypes of two of the lineages differ by a single repeat at just one STR, suggesting very recent shared ancestry, their respective ePAR haplotypes differ greatly, implying that a recent recombination has occurred close to the new boundary (P2/F2 and P3/F3 in Fig 6 and S3 Fig). This recent shared ancestry is also confirmed by the fact that both families have an identical surname that has a low frequency in Belgium (*ca*. 550 carriers in 2008) suggesting a close genealogical relatedness in the patrilineal line [54]. Nonetheless, our minimum recombination estimate is compatible with the sperm CO data for the two intervals surveyed, and suggests that the entire ePAR has a recombination rate of at least six times genome-average (~5.8 cM/Mb, compared with a genome-average male recombination rate of at most 0.9 cM/Mb [55]). The canonical PAR1 supports a male crossover rate seventeen times higher than genome-average and four times greater than the next most recombinogenic region of comparable physical length [56]; our data therefore demonstrate that the ePAR is an active, recombinationally-hot domain in the male germline.

## Discussion

Despite comprising less than 5% of the human Y chromosome, PAR1 plays a fundamental role during male meiosis. Indeed, failure of the human X and Y chromosomes to pair and exchange genetic information within this region is not only associated with paternal inheritance of sex-chromosomal aneuploidy but also intimately linked with male fertility *per se* [5–7, 57, 58]. Our appreciation of the latter has been furthered by mouse studies demonstrating that high levels of achiasmate X and Y trigger a spindle assembly checkpoint resulting in an apoptotic response [59]. Increased infertility in male mice has also been linked with disruption of sequence homology across the mouse PAR [60], demonstrating the importance of the length of sequence identity for successful X-Y pairing. Given the recent discovery that the human PAR1 varies in length among humans [11], we therefore sought to examine the recombination behaviour of this proximally extended 110-kb X-derived ePAR.

We measured recombination activity in the ePAR by directly examining gametic DNA from appropriate sperm donors. Since thousands of sperm can be screened per donor, this approach not only allows efficient estimation of rates (down to 0.0004%, [36]) but can give detailed insight into the dynamics of recombination, even when only one or two men are available for study. Such analyses have been instrumental in establishing that human meiotic recombination, including that in PAR1, is not randomly distributed, but clusters into narrow 1-2-kb-wide intervals, or hotspots [61, 62]. However, this approach, which is based on long PCR, is not easily scalable to even modestly-sized genomic regions such as the ePAR, so here we exploited published human male meiotic DSB maps [19] in order to target tractable sub-regions for bulk sperm analysis. *De novo* recombinants were detected in both sub-regions analysed, and their frequencies, distributions and characteristics were entirely consistent with classic hotspots shaping the recombination landscape of the ePAR. We complemented these sperm data by examining ePAR diversity amongst men of the I2a Y sublineage, estimating that the entire region has a historical recombination frequency of at least six times the male genome average, and thus we conclude that the ePAR very likely contributes to the critical crossover function attributed to the canonical PAR1. Whether this expansion leads to a selective advantage, as proposed for rearrangements altering the mouse PAR (see [60]), remains to be seen.

Sperm DNA approaches have given unprecedented insight into the dynamics of recombination at the sub-kilobase scale, ranging from inter-individual differences in activity [35, 38] through to haplotype-specific differences for a given man [37, 39] but have traditionally relied on pedigree or LD analysis to identify suitable target regions [22,42]. Here, for the first time, we primarily made use of recombination initiation maps to guide our efforts. As noted on a genome-wide scale, the male-specific DSB clusters on the X chromosome relating to the ePAR show reasonable correspondence with LD-based hotspot prediction (6/10 [60%] DSB clusters map to LD hotspots, *cf*. 73% genome-wide, whereas 5/6 [83%] LD hotspots in the region map to DSB clusters, *cf*. 68% genome-wide [19]). Since the LD landscape in this region is dominated by female recombination, this indicates that the chromatin structure of this portion of the X chromosome during prophase I in most males must be very similar to that in females, though of course repair of such DSBs in these non-ePAR carriers must be via the sister chromatid. Since we observe NCOs in both orientations, it seems this 110-kb region probably experiences the same clustering of initiating lesions when embedded on the Y chromosome, and that subsequent spreading of the synaptonemal complex from the canonical PAR1 ensures engagement and repair with whichever homologue is intact.

Although we observed reasonable correspondence with LD hotspot locations, there were some exceptions and it is tempting to speculate that these may be indicative of sex-specific differences in DSB induction. However, as acknowledged by Pratto *et al*., LD-only hotspots could be the consequence of lower-frequency PRDM9 alleles not assessed in their study, and it is possible that DSB clusters could reflect activity that has yet to make an impact at the population level [19]. Alternatively, repair of DSBs to give rise to NCOs exclusively would have extremely localised effects on haplotype diversity and may even go undetected in the absence of suitably located polymorphisms. Recent refined sex-specific genetic maps derived from >100,000 meioses in pedigrees indicate that there are in fact only a few hundred female- or male-specific recombination hotspots throughout the autosomes in comparison to the tens of thousands of total hotspots predicted by LD [63]. On the other hand, sexually dimorphic regions, *i*.*e*. 10-kb intervals with significant sex differences in rate, are observed to be more common by an order of magnitude.

Overall, population-based methods are generally good at predicting hotspot location, as noted here and elsewhere [42], but they do not perform so well in predicting hotspot activity. Certainly there is no consistent relationship between LD breakdown and DSB strength (i.e. DMC1-SSDS signal) in our data, though the latter were ascertained in men unlikely to be ePAR carriers and may therefore be particularly influenced by the lifetime of ssDNA intermediates [64] and/or differences in DMC1 loading [65] since SSDS signal on the strictly sex-specific portions of the X and Y is 3-7x higher than on the autosomes [19]. Our sperm data show comparable rates to those observed at autosomal and PAR1 hotspots and although limited to just two intervals, nonetheless show the expected relative relationship with DSB strength. Future sperm CO+NCO analyses might therefore specifically target the strongest DSB clusters reported by Pratto *et al*. [19] to see if they manifest as hotter than characterized sperm hotspots within the autosomes. Such hotspots would offer the opportunity to recover efficiently even atypical events that might provide further mechanistic insight into human meiotic recombination.

Our study suggests that the haplogroup I2a-associated ePAR is likely to have a more geographically restricted distribution than originally proposed [11]. In the course of identifying carriers we established by junction PCR that the ePAR was present within the two sister I2a sub-lineages I-L1286 and I-L1294, both of which occur predominantly within Northwestern Europe, but was absent from two Hungarian males within the I-M423 sub-haplogroup as determined by resequencing of 3.7 Mb of Y-specific DNA [66] (see S3B Fig). The majority of I-P37.2 men belong to the sub-lineage I-M423, which is predominantly found within Southeastern Europe, and rarely encountered in Northwestern Europe [67], hence its probable absence from the dataset tested by Mensah *et al*. So, whilst we would expect to find haplogroup-I2a ePAR carriers at a frequency of approximately 1% among Northwest European men as originally reported [11], we would expect only a minority of I2a men in Southwest Europe to be carriers of the ePAR.

Breakpoint sequence analysis [11] has shown that the ePAR owes its origin to NAHR between repeated sequences (LTR6B elements), so it is inherently likely to be recurrent. Indeed, its presence in two distinct Y haplogroups shows that it has occurred at least twice. The increasing size of population-based genome-wide SNP datasets, (*e*.*g*. [68]), may allow further examples of the ePAR, or, indeed, other PAR1 extensions, to be identified and characterized. With sufficient numbers of independent occurrences in hand, the influence of sequence diversity of the mediating LTR6B sequences will be able to be understood in detail.

## Methods

### Samples and ethical approvals

North European semen samples were collected with written informed consent, and ethical approval for their use in recombination studies has been granted to CAM by NRES-East Midlands (REC ref. 6659). Sperm DNA was prepared as described in [36]. Additional DNA samples were also collected with written informed consent following University of Leicester ethical review (refs.: maj4-46d9 and maj4-cb66). Blood DNA samples originally analysed in [11] were part of an institutional genome-wide CNV study that was approved by KU Leuven review board (protocol number S55513). Lymphoblastoid cell-line DNA from CEPH family 1334 is available from the Coriell Institute (https://www.coriell.org/).

### Identification of potential ePAR sperm donors and other ePAR carriers

One sperm donor (man 20) was previously identified as carrying a duplication of the X chromosome that encompassed the canonical PAR1 boundary and extended at least 12 kb proximal to this (S1 Fig). Twenty-three Y-STRs were typed in 81 donors, including man 20, using the PowerPlex Y23 kit (Promega). Y-chromosome haplogroups were predicted from the resulting STR haplotypes using a Bayesian Allele Frequency approach (http://www.nevgen.org/). Man 20 and man 53 were predicted to carry the haplogroup I2a-L233 sublineage. Two further unrelated ePAR carriers were found by surveying PowerPlex Y23 data to predict haplogroup I2a Y chromosomes among laboratory collections of DNA samples. A first-generation male from CEPH family 1334 (NA12146) was identified as another candidate carrier; he was reported to have an apparent duplication of X-linked SNPs in the vicinity of the ePAR1 (hg19 chrX:2694151–2808548; hg38 chrX:2776110–2890507) in DGV (http://dgv.tcag.ca/dgv/app/home), and predicted to belong to the same I2a sub-haplogroup based on his Y-STR profile (data kindly provided by C.Tyler-Smith, Wellcome Trust Sanger Institute). We also typed two Hungarian males known from sequencing of 3.4Mb of their male specific Y to have the most distantly related I2a sublineage (I2a-M423) [66] to determine whether all males within I2a possessed an ePAR.

### Confirmation of ePAR status

A duplex PCR consisting of a 848-bp fragment spanning the ePAR junction (*i*.*e*. distal X-specific LTR6B and proximal PAR1-specific LTR6B) together with a 1551-bp control fragment from the *SRY* gene was used to verify the ePAR rearrangement. PCRs were carried out in the buffer described in [69] using primers ePARjunc-F (5´-TGGCAATGTTACTGGAGACG), ePARjunc-R (5´-CAAGGAGTCTGCTGGAAGTC), SRY-F (5´-GGGGTCCCGAGATTTATGTT) and SRY-R (5´-GCTAGAACAAGTTACCCCTC), with an annealing temperature of 60°C and extension temperature of 65°C.

### Confirmation of Y-chromosome haplogroup

A multiplex PCR encompassing nine haplogroup-identifying SNPs within I2a was developed with an annealing temperature of 59°C and extension temperature of 65°C (S5A Table). The resulting products were used in a SNaPshot single-base extension assay using the primer mix detailed in S5B Table according to the manufacturer’s instructions (Thermo Fisher Scientific). The phylogenetic relationships of the haplogroups detected by the SNaPshot assay are shown in S3B Fig.

### Detection of sperm *de novo* recombinants

Assays capable of detecting *de novo* reciprocal crossovers spanning the most distal DSB cluster were designed for each sperm donor following the guidelines in [36]. Similarly, assays able to simultaneously detect reciprocal *de novo* crossovers as well as non-crossover gene conversion events were designed for the proximal target region [36]. Details of the allele-specific primers (ASPs) directed against SNP variants used for recombinant selection are given in S6 and S7 Tables. Phasing of these markers was established empirically using ASP-derived amplicons as templates for allele specific oligonucleotide (ASO) typing [36]. *De novo* recombinants were also characterized by the same method. Details of ASOs are given in S8 Table.

### Sequence analysis of the ePAR

Overlapping long-PCR amplicons were designed to cover the ePAR region (details of the primer pairs are given in S9 Table). The amplicons were pooled equimolar for each individual in two sets, cleaned with Agencourt AMPure XP beads (Beckman Coulter) and used to make individual-specific libraries using the Ion Xpress Library kit and barcodes (Thermo Fisher Scientific) according to the manufacturer’s instructions for 400-bp sequencing. Libraries were size-selected on 1.8% LE agarose, gel-purified using a Zymoclean DNA Recovery kit (Zymo Research), quantified using an Agilent 2100 Bioanalyzer and pooled equimolar. Sequencing templates were prepared using the Ion PGM HI-Q OT2 Kit and sequencing was performed according to manufacturer’s instructions in two runs on an Ion Torrent PGM using the Ion PGM HI-Q Sequencing Kit and 316v2 Chips (Thermo Fisher Scientific). Reads were mapped to the human reference sequence (hg19) using the Torrent Suite Software 5.0.2. The mean number of Q20 bases called per individual across the two runs was 69,342,818 (range: 24,051,513–134,452,533) and mean number of mapped reads was 308,842 (range: 117,148–753,664). Summary statistics for each individual sequenced are shown in S1 Table. See S1 Text for details of validation. The fastq files can be accessed at https://www.ncbi.nlm.nih.gov/sra/SRP155538.

### Phasing of the ePAR

Variant calls were generated by SAMtools 1.3.2 using the bam files and selecting only reads with a minimum mapping quality of 50 and a minimum base quality of 20. The variant calls from the two runs were merged for each individual. Inclusion of female samples and appropriate monochromosomal hybrid cell-line DNA controls (https://www.coriell.org/0/Sections/Collections/NIGMS/Map02.aspx?PgId=496) at the template preparation stage indicated that despite careful design of primer pairs, it was impossible to prevent amplification of portions of Yq11.2; genotype calls for these regions were therefore excluded from further analysis along with Indels and markers mapping to tandemly repetitive sequences. Haplotypes were derived using the program PHASE (http://stephenslab.uchicago.edu/phase/download.html) [48,49], checked for compatibility amongst families and in cases of remaining ambiguity resolved parsimoniously (mean = 7.14 ± 4.40%). See S1 Text for details of validation. Phased X haplotypes over the interval involved in the ePAR translocation were obtained from the CEU and GBR males from the 1000 Genomes Project [34] for comparison.

### TMRCA of haplogroup I2a ePARs

A median-joining Y-STR network of the haplogroup I2a ePARs was constructed using the Network software from Fluxus Engineering [70] and all 23 Y-STRs of the PowerPlex Y23 kit; the bilocal DYS385a,b was included because these Y chromosomes are closely related and the ‘phasing’ issue can be ignored. The TMRCA was estimated from the 23 Y-STR data using the ASD method [51,52] as described in [53], assuming a generation time of 31 yrs [71].

## Supporting information

S1 Fig Duplication of the X chromosome in a North European sperm donor. (PDF)

S2 Fig Features considered when choosing intervals for sperm recombination analysis. (PDF)

S3 Fig Median-joining network of I2a ePAR-carrying males. (PDF)

S1 Table Summary statistics for Ion Torrent sequencing across the ePAR. (PDF)

S2 Table Comparison of ePAR haplotype structures with phase known X chromosomes—SNP markers. (PDF)

S3 Table Comparison of ePAR haplotype structures with phase known X chromosomes—Summary data. (PDF)

S4 Table PowerPlex Y 23 haplotypes for haplogroup I2a ePARs. (PDF)

S5 Table Y-chromosome haplogrouping using a SNaPshot single-base extension assay. (PDF)

S6 Table Primer sequences for sperm recombination analysis. (PDF)

S7 Table Primer combinations and annealing temperatures used for sperm recombination analysis. (PDF)

S8 Table Allele-specific oligonucleotide probe (ASO) sequences. (PDF)

S9 Table Primer sequences for Ion Torrent sequencing templates. (PDF)

S1 Text Validation of Ion Torrent data. (PDF)
