# Supplementary material for: Recombination hotspots in an extended human pseudoautosomal domain predicted from double-strand break maps and characterized by sperm-based crossover analysis
Source: PLoS Genet. 2018 Oct 8;14(10):e1007680. doi: 10.1371/journal.pgen.1007680 (PMC6193736; doi:10.1371/journal.pgen.1007680)
Supplement: S1 Fig — (PDF) [file pgen.1007680.s001.pdf]

S1_Fig. **Duplication of the X chromosome in a North European sperm donor**

A

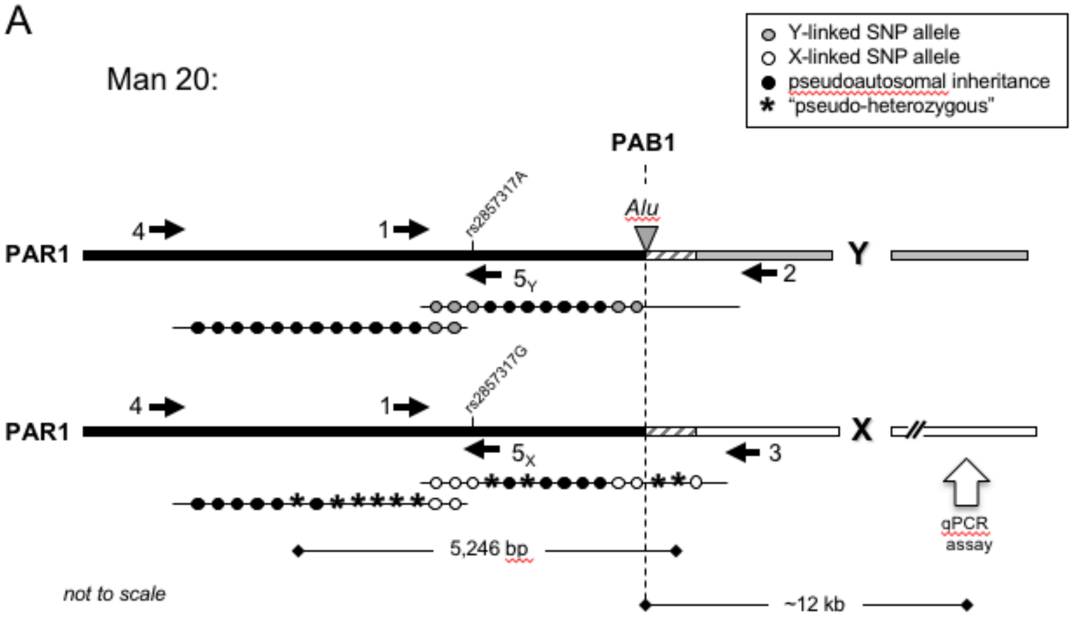

B

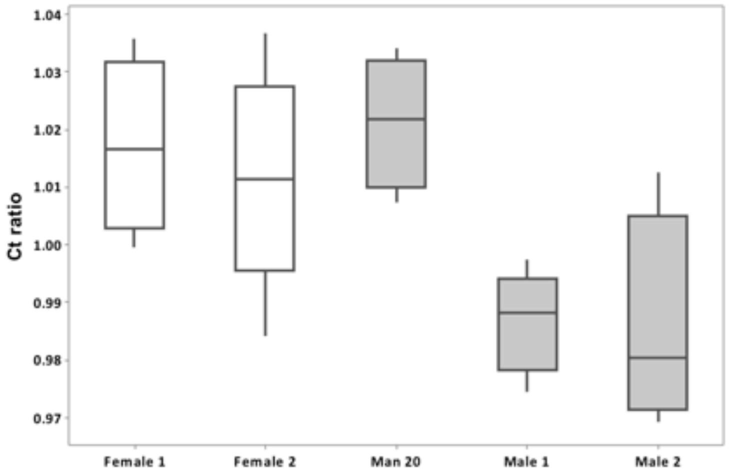

(A) Schematic to show the relative positions of the primer pairs (black arrows) used to generate X- and Y-specific amplicons for SNP haplotyping across the canonical PAR1 boundary (PAB1, which is marked by the insertion site of an *Alu* element on the Y chromosome). Primers 1 and 4 amplify equally well from each of the sex chromosomes, primers 2 and 5_Y are specific to the Y chromosome, whilst primers 3 and 5_X are specific to the X (the 3´ ends of 5_X and 5_Y terminate with SNP rs2857317 alleles G and A respectively). A total of 27 markers were typed by ASO hybridization to these overlapping chromosome-specific amplicons from 50 sperm donors, many showing the expected pseudoautosomal inheritance, but some being sex-linked. Data shown are for man 20; markers with pseudoautosomal inheritance are shown as black circles, Y-linked SNP variants as grey circles and X-linked variants by white circles. Whilst only one SNP variant per marker hybridized to the Y-specific amplicons of this man, surprisingly, both allelic variants at each of ten markers (black asterisks, "pseudo-heterozygous"), in total spanning over 5.2 kb, hybridized to the X-specific amplicons. In an attempt to establish how far this presumed X chromosome duplication extends proximally, a qPCR assay was established ~12 kb from PAB1 (white arrow). (B) The qPCR assay spanning chr X (hg19):2711371-2711465 was carried out on two female and two male DNAs in addition to that from man 20 using qPCR MasterMix Plus for SYBR Green I without UNG (Eurogentec). Reactions were carried out in quadruplicate with inputs ranging from 0.125 to 4 ng, and with 4 to 8 inputs per sample. The data were normalized using a published assay for the estrogen receptor located on chromosome 6 ((hg19):152265487-152265570 [1]). The Ct ratios for man 20 were more similar to those of the two females than the two males as shown by the boxplots. These results suggested the X chromosome duplication in man 20 covers at least 17 kb.

1. Mhlanga MM and Malmberg L (2001) Using molecular beacons to detect single-nucleotide polymorphisms with real-time PCR. Methods 25: 463-471
