# Supplementary material for: Recombination hotspots in an extended human pseudoautosomal domain predicted from double-strand break maps and characterized by sperm-based crossover analysis
Source: PLoS Genet. 2018 Oct 8;14(10):e1007680. doi: 10.1371/journal.pgen.1007680 (PMC6193736; doi:10.1371/journal.pgen.1007680)
Supplement: S2 Fig — (PDF) [file pgen.1007680.s002.pdf]

S2_Fig: **Features considered when choosing intervals for sperm recombination analysis**

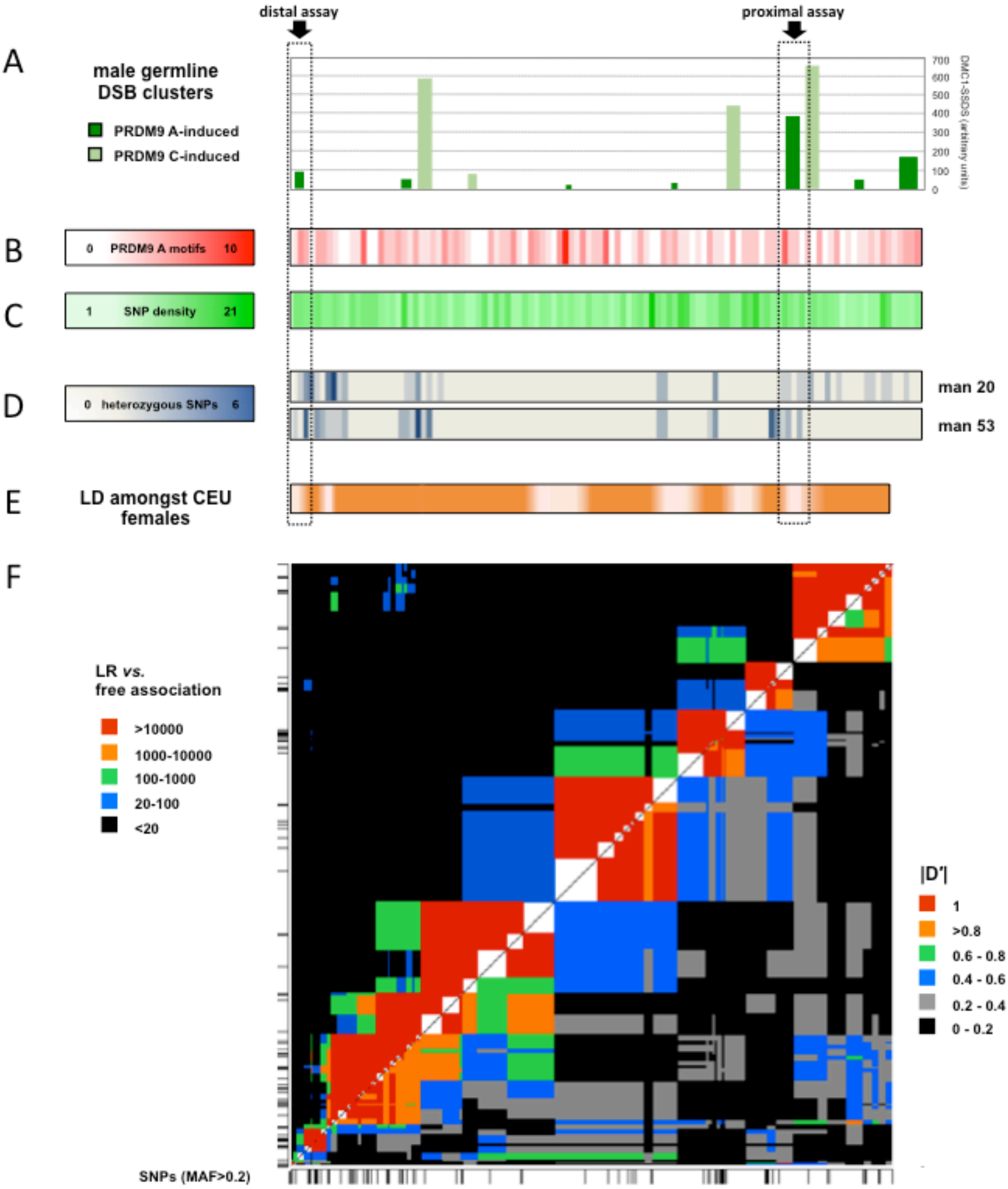

(A) Distribution and intensity of DSB clusters that fall within the X-derived portion of the ePAR. Clusters were defined by anti-DMC1 SSDS using testis biopsies from five men and designated as being induced by PRDM9 A (dark green) or PRDM9 C (light green); strength is shown as the mean across relevant individuals using the arbitrary values reported in the original work [1]. (B) The occurrence of both near (7/8) and complete (8/8) matches to the degenerate PRDM9 A hotspot motif CCNCCNTNNCCNC [2], found by regular expressions using Perl, are shown per 1-kb interval in the upper panel (shades of red). (C) Similarly, the density of SNPs was calculated from dbSNP build 150 and is shown in the lower panel (shades of green). (D) Frequency of heterozygous SNP markers per 1-kb interval identified in each of the two ePAR-positive sperm donors (man 20, man 53) as determined by Ion Torrent sequencing. (E) Linkage disequilibrium (LD) heat map derived from the 50 CEU females from the 1000 Genomes Project [3] (more intense orange, the stronger the LD). Data are based on Lewontin's |D´| values [4] which are normalised values of the corresponding LD coefficient D (the latter simply being the difference in observed versus expected frequencies of a two-SNP haplotype within a population sample). Normalisation which takes into account the minor allele frequencies (MAF) of each of the SNP pair, allows direct comparison of |D´| values between different marker pairs. |D´| values of 1.0 by definition indicate marker pairs that are in complete LD. Only SNPs with MAF >0.2 that passed tests for Hardy-Weinberg equilibrium specifically derived for markers on the X chromosome [5] were considered here; these stringent criteria meant that LD corresponding to the most proximal portion of the ePAR interval could not be examined. (F) Lower right-hand of the diagonal shows the underlying pairwise measures of |D´| between SNPs (tick marks on the axes) for track shown above; it is colour-coded as indicated to the right of the plot. The associated likelihood ratio (LR) versus free association for each comparison is shown in the upper left, colour-coded as indicated to the left; for example, a pairwise comparison shown in red indicates that the two markers concerned are 10,000 times more likely to be in linkage disequilibrium than they are in free association. Points in this graphic are plotted as rectangles centred on each SNP and extending halfway to adjacent markers. The upper and lower plots show strong concordance indicating robust interpretation. There are six regions of LD breakdown ranging in size from 1,948 bp to 11,013 bp (median 5,956 bp). Based on the data presented in (A) to (F), one distal and one proximal interval were chosen for sperm recombination assay development as shown by arrows and dotted boxes at the top of the figure.

1. Pratto F, Brick K, Khil P, Smagulova F, Petukhova GV and Camerini-Otero RD (2014) Recombination initiation maps of individual human genomes. Science 346: 1256442.
2. Myers S, Freeman C, Auton A, Donnelly P and McVean G (2008) A common sequence motif associated with recombination hot spots and genome instability in humans. Nature Genet 40:1124-1129.
3. Consortium GP (2012) An integrated map of genetic variation from 1,092 human genomes. Nature 491: 56-65.
4. Lewontin RC (1964) The interaction of selection and linkage. I. General considerations; Heterotic models. Genetics 49:49-67
5. Graffelman J and Weir B (2016) Testing for Hardy–Weinberg equilibrium at biallelic genetic markers on the X chromosome. Heredity 116: 558-568.
