# Supplementary material for: Recombination hotspots in an extended human pseudoautosomal domain predicted from double-strand break maps and characterized by sperm-based crossover analysis
Source: PLoS Genet. 2018 Oct 8;14(10):e1007680. doi: 10.1371/journal.pgen.1007680 (PMC6193736; doi:10.1371/journal.pgen.1007680)
Supplement: S3 Fig — (PDF) [file pgen.1007680.s003.pdf]

S3_Fig. **Median-joining network of I2a ePAR-carrying males**

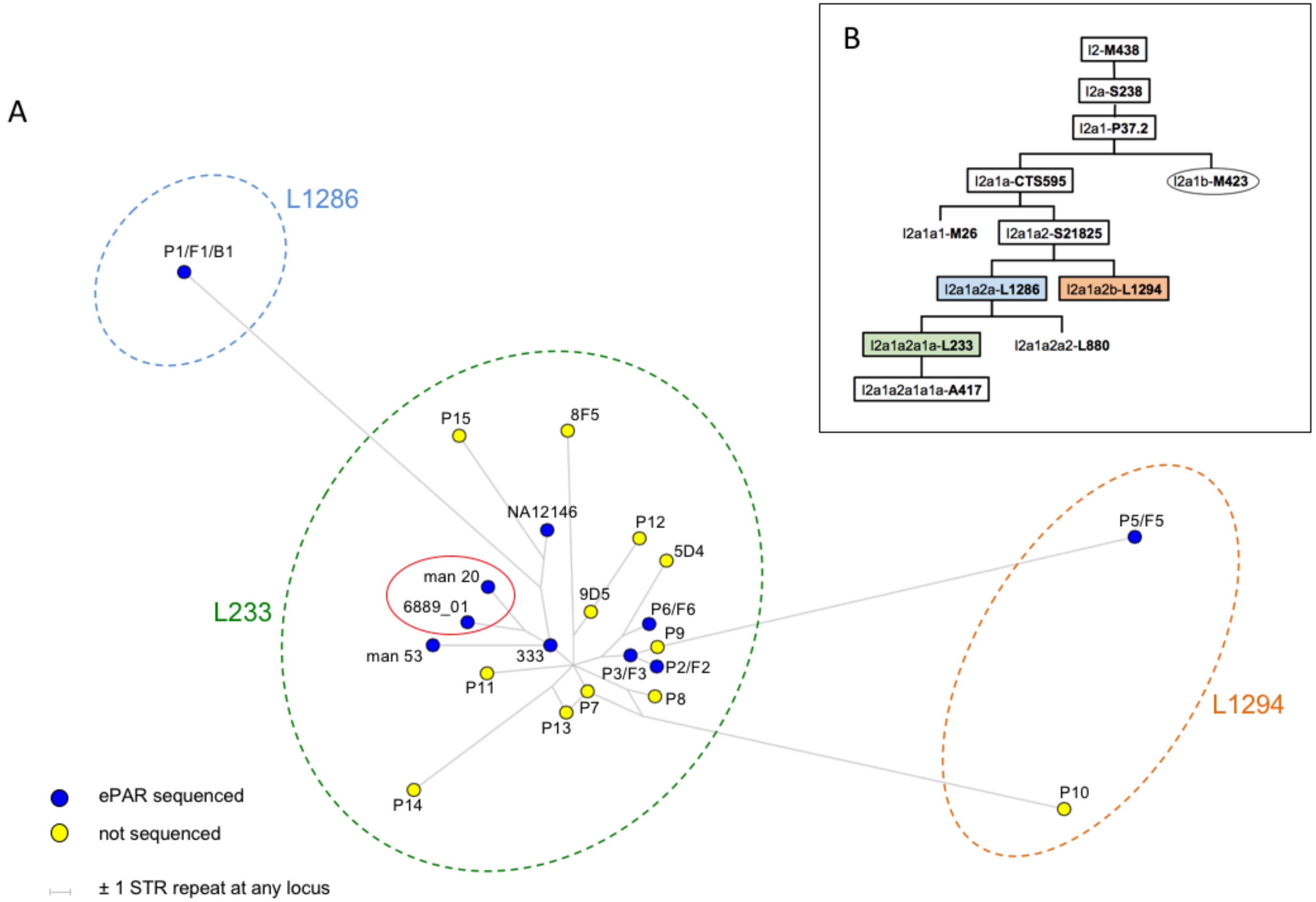

(A) A network was constructed using the 23-Y-STR haplotype for each of 22 unrelated men verified as carrying the ePAR by junction-specific PCR and predicted to belong to haplogroup I2a (blue circles, ten ePAR sequenced haplotypes; yellow circles, further twelve that were not sequenced, see also S6 Table). All the nodes are joined by links with lengths proportional to the number of single-step STR length changes. The red ellipse encircles the two men who share the modal ePAR haplotype as shown in Figs 5 and 6. The central cluster of ePARs (enclosed within the green dashed line) share the Y sub-haplogroup I-L233 whilst the outliers belong to sub-haplogroups I-L1286 (blue dashed line) and I-L1294 (orange dashed line). (B) The panel in the top right shows the SNP haplogroup tree for the I2a lineage using the ISOGG 2018 nomenclature (https://isogg.org/tree/); the markers included in the SNaPshot® single-base extension assay (see Methods and S8 Table) are shown in boxes. Sub-haplogroups established for the 28 ePARs sampled are colour-coded as in (A). Note that since the defining SNP for I-L880 was not typed, it is possible that P5/F5 may actually belong to this sublineage rather than the underived I-L1286.
