# Supplementary material for: Recombination hotspots in an extended human pseudoautosomal domain predicted from double-strand break maps and characterized by sperm-based crossover analysis
Source: PLoS Genet. 2018 Oct 8;14(10):e1007680. doi: 10.1371/journal.pgen.1007680 (PMC6193736; doi:10.1371/journal.pgen.1007680)
Supplement: S1 Table — (PDF) [file pgen.1007680.s004.pdf]

S1_Table. **Summary statistics for Ion Torrent sequencing across the ePAR**

individual			bases	>=Q20 bases	>=Q20 bases, %	reads	mapped reads	% of reads mapped
**NA10847**	*female*	da	43,979,848	39,307,954	89.38	193,337	179,808	93.00
**NA12146**	*ePAR*	fa	71,728,327	64,684,810	90.18	290,574	271,962	93.59
**NA12239**	*female*	mo	86,728,887	77,742,730	89.64	359,398	334,770	93.15
**man 20**	*ePAR*		78,320,031	70,679,322	90.24	316,557	306,178	96.72
**man 53**	*ePAR*		72,216,306	65,159,010	90.23	284,570	279,360	98.17
**B1 ***	*ePAR*	br	66,763,804	59,929,318	89.76	264,729	232,026	87.65
**P1 ***	*ePAR*	pa	66,218,114	59,721,814	90.19	262,450	235,900	89.88
**F1 ***	*ePAR*	fa	80,785,440	72,804,184	90.12	336,278	307,895	91.56
**P2 ***	*ePAR*	pa	78,977,048	70,732,103	89.56	325,234	310,205	95.38
**F2 ***	*ePAR*	fa	36,711,977	32,891,346	89.59	163,180	157,325	96.41
**P3 ***	*ePAR*	pa	149,637,283	134,452,533	89.85	810,574	753,664	92.98
**F3 ***	*ePAR*	fa	84,790,584	75,859,154	89.47	345,952	320,717	92.71
**P4 ***	*ePAR*	pa	85,253,719	76,863,868	90.16	355,542	347,721	97.80
**F4 ***	*ePAR*	fa	85,659,925	77,183,601	90.10	343,893	335,654	97.60
**P5 ***	*ePAR*	pa	26,747,753	24,051,513	89.92	118,408	117,148	98.94
**F5 ***	*ePAR*	fa	85,472,838	76,732,964	89.77	346,425	321,536	92.82
**P6 ***	*ePAR*	pa	113,795,421	101,829,085	89.48	476,539	457,852	96.08
**F6 ***	*ePAR*	fa	75,312,769	66,838,973	88.75	300,658	276,314	91.90
**6689_01**	*ePAR*		83,255,363	75,124,261	90.23	333,594	321,682	96.43
**333**	*ePAR*		72,691,290	64,267,808	88.41	320,268	309,115	96.52
		min	**26,747,753**	**24,051,513**	**88.41**	**118,408**	**117,148**	**87.65**
		max	**149,637,283**	**134,452,533**	**90.24**	**810,574**	**753,664**	**98.94**
		mean	**77,252,336**	**69,342,818**	**89.75**	**327,408**	**308,842**	**94.46**

* from Mensah *et al.* 2014 PLoS genetics, 10: e1004578.
da = daughter, fa=father, mo=mother, br=brother, pa=patient
