# Supplementary material for: Recombination hotspots in an extended human pseudoautosomal domain predicted from double-strand break maps and characterized by sperm-based crossover analysis
Source: PLoS Genet. 2018 Oct 8;14(10):e1007680. doi: 10.1371/journal.pgen.1007680 (PMC6193736; doi:10.1371/journal.pgen.1007680)
Supplement: S2 Table — (PDF) [file pgen.1007680.s005.pdf]

S2_Table: **Comparison of ePAR haplotype structures with phase-known X chromosomes – SNP markers**

**SNP ID (chr X)**	**hg19 (chr X)**	**SNP ID**	**hg19 (chr X)**	**SNP ID**	**hg19 (chr X)**	**SNP ID**	**hg19 (chr X)**	**SNP ID**	**hg19**
rs28579419	2699645	rs311182	2714277	rs5982871	2739032	rs142753289	2772820	rs5939380	2784051
rs60075487	2699676	rs5939326	2714531	rs6641652	2740681	rs4892899	2773521	rs200259684	2784879
rs2306737	2699968	rs141584360	2714584	rs3828931	2740891	rs4892900	2773780	rs6642050	2785428
rs2306736	2700027	rs4484858	2715425	rs3828930	2740892	rs5939133	2774700	rs62582323	2785712
rs5939319	2700157	rs2316291	2717033	rs73190969	2741078	rs4993468	2774793	rs62582325	2785737
rs5939320	2700202	rs311189	2717067	rs141205366	2741322	rs7473759	2774990	rs62582327	2785740
rs5982852	2702047	rs5939327	2717234	rs185534666	2741406	rs7473760	2775002	rs62582329	2785749
rs5982853	2702143	rs311190	2717538	rs6641656	2748292	rs148979483	2775148	rs190308716	2785936
rs111595179	2702339	rs5939329	2718135	rs5939348	2748421	rs58163192	2775211	rs12558009	2786029
rs5982854	2702568	rs752782071	2718189	rs5939350	2749521	rs141405035	2775259	rs6642051	2786038
rs73433431	2702698	rs11152547	2724389	rs5939351	2750122	rs9781871	2775356	rs73435434	2792662
rs5982855	2702799	rs7058222	2724429	rs2873118	2750768	rs905375	2775601	rs75069845	2792838
rs5982856	2702946	rs7062707	2724448	rs6641657	2751904	rs5939134	2778322	rs6567674	2794461
rs5982584	2703354	rs5939335	2725660	rs12396748	2752070	rs2316285	2778433	rs7065465	2794858
rs1486175	2703391	rs189287974	2727265	rs12387509	2752333	rs1905995	2778526	rs5982604	2797795
rs1419931	2703633	rs6567647	2728221	rs5982590	2752707	rs5939374	2778546	rs5982605	2797895
rs6641645	2704335	rs12010750	2728516	rs113160927	2753182	rs1905996	2778587	rs12388511	2798480
rs6642018	2704808	rs141066369	2728872	rs7057656	2753527	rs5939375	2778715	rs1269	2800624
rs6642019	2704989	rs5982866	2729011	rs138650022	2753713	rs5982900	2778796	rs1268	2800677
rs113922957	2705011	rs5939336	2729281	rs7892329	2754793	rs1905997	2778832	rs9355	2800788
rs112589751	2705265	rs5982867	2729346	rs5982876	2756413	rs10871869	2778982	rs12844789	2801000
rs5982858	2705462	rs12851007	2732634	rs5982877	2757233	rs10871870	2778996	rs12859113	2801026
rs5982859	2706340	rs5939124	2733109	rs5982878	2757399	rs11152551	2779171	rs211664	2801155
rs5982860	2706487	rs5939125	2733210	rs5982879	2757754	rs11152552	2779211	rs60445074	2801158
rs5939117	2707060	rs3672	2733641	rs4892896	2757783	rs10871871	2779330	rs12391908	2802182
rs5939324	2707142	rs3671	2733668	rs11152548	2759615	rs2306735	2779570	rs62582348	2802356
rs311166	2707978	rs5939340	2734838	rs62582317	2767637	rs4892901	2780265	rs211665	2802368
rs145903180	2709600	rs5939341	2734930	rs145268586	2767957	rs4892902	2780319	rs1637783	2802396
rs112470161	2710504	rs140285516	2735299	rs2316287	2768611	rs5939135	2780533	rs1637784	2802397
rs311167	2710506	rs5939342	2735539	rs5939358	2768713	rs6642045	2780747	rs211668	2802726
rs311168	2710840	rs5939343	2735621	rs9320050	2768851	rs6642046	2780826	rs76495523	2802915
rs311169	2710995	rs5939344	2735741	rs9320051	2768864	rs6642047	2780829	rs59272600	2802991
rs311170	2711429	rs146579399	2735895	rs6641662	2769103	rs149865569	2780978	rs5939140	2803276
rs4892892	2711722	rs6642031	2735926	rs62582319	2769284	rs12391316	2781220	rs56019734	2803303
rs2291380	2712283	rs5939126	2736196	rs5939360	2769334	rs7057853	2781260	rs5939141	2803387
rs311174	2713012	rs1809566	2736301	rs7058332	2771314	rs55776760	2781357	rs5939142	2803469
rs1639329	2713069	rs2316290	2736503	rs5939361	2771315	rs5939376	2782305	rs5939143	2803688
rs1639330	2713073	rs6567653	2737097	rs5939362	2771540	rs11152553	2782384	rs5939386	2803689
rs311175	2713089	rs2018620	2737149	rs4892898	2772275	rs5939377	2782455	rs5018317	2804185
rs7061550	2713211	rs5982869	2737194	rs7879795	2772660	rs5939136	2782633	rs211654	2805786
rs311177	2713698	rs901321	2737282	rs7882856	2772694	rs5939378	2783107	rs211655	2806196
rs311179	2713763	rs5939128	2737851	rs6567664	2772772	rs5939137	2783555		
rs311180	2713807	rs5982870	2738809	rs6567665	2772792	rs5939379	2783776		

NOTE: To lift over to hg38 add 81959 to hg19 co-ordinate
