# Supplementary material for: Recombination hotspots in an extended human pseudoautosomal domain predicted from double-strand break maps and characterized by sperm-based crossover analysis
Source: PLoS Genet. 2018 Oct 8;14(10):e1007680. doi: 10.1371/journal.pgen.1007680 (PMC6193736; doi:10.1371/journal.pgen.1007680)
Supplement: S3 Table — (PDF) [file pgen.1007680.s006.pdf]

S3_Table**: Comparison of ePAR haplotype structures with phase-known X chromosomes - summary data**

		block A	block B	block C	block D	block E	block F	block G	block H	bloc[illegible] I
	no. SNPs:	6	47	11	29	19	29	41	7	
	hg19 (chr X) start:	2699645	2702047	2724389	2732634	2748292	2767637	2778322	2792662	2800[illegible]
	hg19 (chr X) end:	2700202	2718189	2729346	2741406	2759615	2775601	2786038	2798480	2806[illegible]
**no. different haplotypes amongst:**										
I2a ePAR (n=10)		2	7	2	3	1	4	4	2	
R1b ePAR (n=1)		1	1	1	1	1	1	1	1	
CEU (n=49)		5	20	5	9	4	13	11	6	
GBR (n=46)		6	17	5	6	4	11	9	5	
**no. unique haplotypes amongst:**										
I2a ePAR (n=10)		0	4	0	2	0	2	1	0	
R1b ePAR (n=1)		0	1	0	0	0	1	0	0	
CEU (n=49)		0	12	2	5	0	6	4	3	
GBR (n=46)		1	9	2	2	1	5	2	5	
**% I2a ePAR shared with:**										
CEU		100	54.5	100	81.8	100	72.7	90.9	100	9[illegible]
GBR		100	54.5	100	81.8	100	72.7	90.9	100	9[illegible]
**% CEU haplotypes shared with:**										
all ePARs		69.4	49.0	89.8	55.1	67.3	16.3	69.4	55.1	4[illegible]
GBR		100	71.4	91.8	89.8	100	81.6	91.8	83.7	7[illegible]
**% GBR haplotypes shared with:**										
all ePARs		73.9	45.7	89.1	45.7	60.9	17.4	50.0	80.4	6[illegible]
CEU		97.8	76.1	91.3	93.5	97.8	84.8	84.8	91.3	8[illegible]

Adjacent blocks of markers are separated by clusters of mapped meiotic DSBs as reported in Pratto *et al*. 2014 *Science* 346: 1256442.

NOTE: To lift over to hg38 add 81959 to hg19 co-ordinate
