# Supplementary material for: Recombination hotspots in an extended human pseudoautosomal domain predicted from double-strand break maps and characterized by sperm-based crossover analysis
Source: PLoS Genet. 2018 Oct 8;14(10):e1007680. doi: 10.1371/journal.pgen.1007680 (PMC6193736; doi:10.1371/journal.pgen.1007680)
Supplement: S4 Table — (PDF) [file pgen.1007680.s007.pdf]

S4_Table. **PowerPlex Y-23 STR haplotypes for haplogroup I2a carriers of ePAR.**

	DYS393	DYS390	DYS19	DYS391	DYS385a	DYS385b	DYS439	DYS389I	DYS392	DYS389II	DYS458	DYS437	DYS448	YGATAH4	DYS456	DYS576	DYS570	DYS438	DYS481	DYS549	DYS533	DYS635	DYS643
**NA12146**	13	22	15	10	12	15	11	13	11	29	18	14	17	11	14	18	17	10	27	12	13	21	12
**man 20**	13	22	15	10	12	15	11	14	11	31	18	14	18	11	14	17	17	10	28	12	13	21	12
**6689_01**	13	23	15	10	12	15	11	14	11	30	19	14	18	11	14	17	18	10	28	12	13	21	12
**333**	13	23	15	10	12	15	11	14	11	30	18	14	18	11	14	17	17	10	27	12	13	21	12
**man 53**	13	23	16	10	12	15	11	14	11	30	17	14	18	11	14	17	19	10	27	12	13	21	12
**P2/F2***	13	23	15	10	12	15	12	14	12	30	18	14	19	11	14	17	17	10	27	12	12	21	12
**P3/F3***	13	23	15	10	12	15	12	14	11	30	18	14	19	11	14	17	17	10	27	12	12	21	12
**P6/F6***	13	23	15	10	12	15	12	14	11	30	18	14	18	10	14	17	17	10	27	11	12	21	12
**P1/F1/B1***	13	23	15	10	15	16	11	12	11	28	16	14	20	11	14	19	17	10	23	13	12	21	12
**P5/F5***	14	25	16	10	11	14	13	14	11	30	18	16	21	12	15	18	18	10	25	11	12	21	12
**P14***	13	25	15	10	13	16	12	14	11	30	18	14	18	11	14	17	19	10	26	12	13	21	12
**P15***	13	22	15	10	12	15	11	13	11	29	19	14	18	11	13	18	19	10	27	12	12	21	12
**P7***	13	23	15	11	12	15	12	14	11	30	18	14	18	11	14	17	17	10	27	12	13	21	12
**P8***	12	23	15	10	12	14	12	14	11	30	18	14	18	11	14	18	17	10	27	12	13	21	12
**P9***	13	23	16	10	12	15	12	14	11	30	18	14	19	11	14	17	17	10	27	12	12	21	12
**P11***	13	23	16	10	12	15	13	14	11	30	18	14	18	11	14	17	17	10	27	12	13	22	12
**P12***	13	23	15	10	12	15	12	14	11	30	19	14	18	11	14	16	16	10	25	12	13	21	12
**P13***	13	24	15	11	12	15	12	14	11	30	18	14	18	11	14	17	17	10	27	12	13	21	12
**P10***	13	23	15	11	11	14	12	13	11	29	18	16	20	11	14	19	17	10	24	11	14	21	15
**5D4**	13	23	15	10	12	15	12	14	11	30	19	14	18	11	14	17	18	10	27	11	12	21	13
**8F5**	13	23	15	10	12	16	13	13	11	29	19	14	18	10	14	17	18	10	27	12	13	20	11
**9D5**	13	23	15	10	12	15	12	14	11	30	19	14	18	11	14	16	17	10	27	12	13	21	12

* data from Mensah *et al.* 2014 PLoS genetics, 10: e1004578.
