# Supplementary material for: Recombination hotspots in an extended human pseudoautosomal domain predicted from double-strand break maps and characterized by sperm-based crossover analysis
Source: PLoS Genet. 2018 Oct 8;14(10):e1007680. doi: 10.1371/journal.pgen.1007680 (PMC6193736; doi:10.1371/journal.pgen.1007680)
Supplement: S5 Table — (PDF) [file pgen.1007680.s008.pdf]

S5_Table: **Y-chromosome haplogrouping using a SNaPshot® single-base extension assay**

A) Primer sequences and annealing temperature for haplogroup-I2a multiplex

Primer name	5' to 3' sequence	Location of 5' nt in hg19 (chrY)	Location of 5' nt in hg38 (chrY)	% in primer mix	T_m (°C)
I2F1	CTAGTGGCTGCATAAGGTAG	16638691	14526811	6.12	59
I2R1	GTCAGTCAAAAGCTCCTGTC	16638914	14527034		
I2F2	GGAGTCACATCACTTAGGTG	7879298	8011257	8.16	
I2R2	CTTTTCTAGGACCAGACCAC	7879507	8011466		
I2F3	GAGACAAGCATAGTGATAGGG	14491650	12379847	4.08	
I2R3	GGCATGCCCAACTCCTCTTC	14491815	12380012		
I2F4/3	CTGAAGCCTGGGTGTGACTTG	6873997	7005956	6.12	
I2R4/2	GTTCTGGGCTGATATTTCTGC	6874152	7006111		
I2F5	GGGGTGTAGTTTAGACATC	19126608	17014728	14.29	
I2R5	GGCTGAGATTCTATCCTGAG	19126738	17014858		
I2F6	CAGCACTTGTCTTCTGTTTGC	21778604	19616718	10.21	
I2R6	GTGGAGATGGTAAGTTGTCC	21778760	19616874		
I2F8	GGTGATTGATAGCTAGACAGC	14487267	12366536	14.29	
I2R8	GGATGACAGACTCTATGTG	14487496	12366765		
I2F9	CATGGGGAACAGGCAGTGAA	8846879	8978838	8.16	
I2R9	GAACTTCCTTCCTCTCCAAC	8847138	8979097		
I2F10/2	CTATCAGGTAGGCAGAGTG	2887182	3019141	28.57	
I2R10	TCCTAAAGAGTGCAGAGCTG	2887454	3019413		

B) Extension primer sequences for haplogroup-I2a SNaPshot® assay

SNP	hg19 (chrY)	hg38 (chrY)	change	YCC Hg	ISOGG 2018 Hg	5' to 3' primer sequence	% in mix
M438, P215	16,638,804	14,526,924	A to G	I2	I2	AAAGCCTGGAATGTAGACTAATGGT	5.36
S238, L460	7,879,415	8,011,374	A to C	approx. hg: I-P215	I2a	AAAAAAAAAAAAAAAAAAAAAAAAAAAAAAAAAAAAAAA<br>AAAAAACTTGGCTCTGCCTACAGAG	14.29
P37	14,491,684	12,379,881	T to C	D2 and I2a	I2a1	AAAAAAAAAAAAGATAGGGTGGGATTGGTTCA	5.36
CTS595	6,874,115	7,006,074	C to T	I2a-P37.2	I2a1a	AAAAAAAAAAAAAAAAAAAAAAAAACTGCCAACAAATTA<br>AATACC	5.36
S21825	19,126,655	17,014,775	G to A	*not listed*	I2a1a2	AAAAAAAAAAAAAAAAAAAAATGGACTTAGGGAACTTC<br>C	10.71
L1286	21,778,662	19,616,776	G to A	approx. hg: I-L233	I2a1a2a	AAAAAAAAAAAAAAAAAAAAAAAAAAAAAAAAAAAAAAA<br>AAAAAAAAAAAAGTCTGAATGTCTCAAACTC	7.14
L233	14,487,362	12,366,631	G to A	I2a3	I2a1a2a1a	AAAAAAAAAAAAAAAAAAAAAAAAAAAAAAAAAGCTGAT<br>TGATAGCTAGAT	16.07
A417	8,846,964	8,978,923	T to C	I-A417	I2a1a2a1a1a	AAAAAAAAAAAAAAAAAAAAAAAAAAAAAAAAAAAAAAA<br>TTTTTTATACCAAAGCTT	14.29
L1294	2,887,401	3,019,360	T to C	I2a-S21825	I2a1a2b	AAAAAAAAAAAAAAAAAAAAAAAAAAAAAAAAAAAAAAA<br>AAAAAAAAAAAAAAAAAAAAAATTAGCCATTTTTTGTG	21.42
