# Supplementary material for: Recombination hotspots in an extended human pseudoautosomal domain predicted from double-strand break maps and characterized by sperm-based crossover analysis
Source: PLoS Genet. 2018 Oct 8;14(10):e1007680. doi: 10.1371/journal.pgen.1007680 (PMC6193736; doi:10.1371/journal.pgen.1007680)
Supplement: S6 Table — (PDF) [file pgen.1007680.s009.pdf]

S6_Table: **Primer sequences for sperm recombination analysis**

Primer name	5' to 3' sequence	Location of 5' nt in hg19 (chrX)	Location of 5' nt in hg38 (chrX)
X25/56F	GGAAGGTATATAAGCTCTGG	2699568	2781527
9.5F C/A	GATAAATGCACACACATATC/A	2699536	2781495
X9.6F G/T	CCTTCTCCCTGTAACAGG/T	2699628	2781587
9.9F A/G	TTTTTAACCAGACAATGGCA/G	2699949	2781908
X9980F	ACACCGTGGGCAGCAAATCA	2699979	2781938
X11131F	GGACACTTTGCATTATCATC	2701130	2783089
X2.77R	AACATAGCCATGATCCGCAG	2702217	2784176
X12654F	GCGAGATCCTTTAAGATGGG	2702653	2784612
X13778R	CTGGGCAAGAACACTGGTTA	2703777	2785736
X14598R	GTGGCTCACACCTGTAATCCC	2704597	2786556
14.6R T/C	TTACTTTCAAGACAACATGA/G	2704628	2786587
X14763R	TTCTTAAAGGCCCCATCTCC	2704762	2786721
14.8R T/C	TTATTAGGATACTAGTCCTA/G	2704827	2786786
15.0R C/T	GCTGTCACAGAACACCATAG/A	2705030	2786989
15.2R C/T	CTTAGTCCATTTCAGGCTG/A	2705283	2787242
X15423R	AACCTCCAACTGCATTGATTC	2705422	2787381
93.1F A/G	CCATGCCTGGCTATTAAAAA/G	2783088	2865047
X93213F	GAGGGATTTCCACGGATTG	2783212	2865171
93.5F G/T	GATATGTAGCTTCGTGTTTGT/G	2783535	2865494
X94556F	GAGACTAGGAAAGGTGGG	2784555	2866514
X95211F	GAGCGTACTTTGTTTAGGGT	2785210	2867169
X95737R	CTCCCATTCCTGAATCTCTC	2785736	2867695
X98092R	ACTAGAAGACAAAACGCTGG	2788091	2870050
X98641R	GTAGACTGAAGTATGGGGAG	2788640	2870599
X99925F	CACCACCTCCAGCAGTAATAA	2789924	2871883
X100924R	GTCCTGGATTTGAATACTG	2790923	2872882
102.6R A/G	CAGTGGCCTACACAGATAT/C	2792680	2874639
X102760R	TCCAGTTCATTCTCCTAATC	2792759	2874718
102.8R A/G	ATCCCAGAGATTTTGAGTGAT/C	2792858	2874817

The alternative 3’ nucleotides of allele-specific primers (ASPs) are shown in red.
