# Supplementary material for: Recombination hotspots in an extended human pseudoautosomal domain predicted from double-strand break maps and characterized by sperm-based crossover analysis
Source: PLoS Genet. 2018 Oct 8;14(10):e1007680. doi: 10.1371/journal.pgen.1007680 (PMC6193736; doi:10.1371/journal.pgen.1007680)
Supplement: S7 Table — (PDF) [file pgen.1007680.s010.pdf]

S7_Table: **Primer combinations and annealing temperatures used for sperm recombination analysis**

Assay	Man	Primer combination	T_m (°C)	Purpose
Distal	20	9.5F C + X2.77R<br>9.5F A + X2.77R	58<br>56	phasing of 5' selector sites
Distal	20	X11131F + 14.8R C<br>X11131F + 14.8R T	54<br>54	phasing of 3' selector sites
Distal	20	9.5F C + 14.8R C	57-55	crossover detection (1° PCR) orientation A
Distal	20	X9.6F G + 14.6R C	54	crossover detection (2° PCR) orientation A
Distal	20	9.5F A + 14.8R T	56-54	crossover detection (1° PCR) orientation B
Distal	20	X9.6F T + 14.6R T	54	crossover detection (2° PCR) orientation B
Distal	20	X9980F + X14598R	64	crossover detection (3° PCR) orientation A & B
Distal	53	9.5F C + X13778R<br>9.5F A + X13778R	59<br>58,57	phasing of 5' selector sites
Distal	53	X12654F + 15.2R C<br>X12654F + 15.2R T	59<br>59	phasing of 3' selector sites
Distal	53	9.5F A + X15423R	56	crossover detection (1° PCR #1) orientation A
Distal	53	X25/56F + 15.2R C	58	crossover detection (1° PCR #2) orientation A
Distal	53	9.9F G + 15.0R C	61	crossover detection (2° PCR) orientation A
Distal	53	9.5F C + X15423R	56	crossover detection (1° PCR #1) orientation B
Distal	53	X25/56F + 15.2R T	58	crossover detection (1° PCR #2) orientation B
Distal	53	9.9F A + 15.0R T	61	crossover detection (2° PCR) orientation B
Distal	53	X9980F + X13778R	61	crossover detection (3° PCR) orientation A & B
Proximal	20	X94556F + 102.8R A<br>X94556F + 102.8R G	60, 58	phasing (1° PCR)
Proximal	20	X95211F + X100924R	57	phasing (2° PCR 5’ amplicon)
Proximal	20	X99925F + X102760R	58	phasing (2° PCR 3’ amplicon)
Proximal	20	X94556F + 102.8R A	60, 58	recombinant detection (1° PCR) orientation A
Proximal	20	X95211F + 102.6R A	61, 60	recombinant detection (2° PCR) orientation A
Proximal	20	X94556F + 102.8R G	60, 58	recombinant detection (1° PCR) orientation B
Proximal	20	X95211F + 102.6R G	59	recombinant detection (2° PCR) orientation B
Proximal	53	93.1F A + X98641R<br>93.1F G + X98641R	60-58	phasing (1° PCR)
Proximal	53	X93213F + X95737R	58	phasing (2° PCR 5’ amplicon)
Proximal	53	X95211F + X98092R	58	phasing (2° PCR 3’ amplicon)
Proximal	53	93.1F A + X98641R	60-58	recombinant detection (1° PCR) orientation A
Proximal	53	93.5F T + X98092R	62	recombinant detection (2° PCR) orientation A
Proximal	53	93.1F G + X98641R	60-58	recombinant detection (1° PCR) orientation B
Proximal	53	93.5F G + X98092R	62	recombinant detection (2° PCR) orientation B

For primer details see Table S9.
