# Supplementary material for: Recombination hotspots in an extended human pseudoautosomal domain predicted from double-strand break maps and characterized by sperm-based crossover analysis
Source: PLoS Genet. 2018 Oct 8;14(10):e1007680. doi: 10.1371/journal.pgen.1007680 (PMC6193736; doi:10.1371/journal.pgen.1007680)
Supplement: S8 Table — (PDF) [file pgen.1007680.s011.pdf]

S8_Table: **Allele-Specific Oligonucleotide probe (ASO) sequences**

Assay	Man	ASO name	5' to 3' sequence	Location of SNP in hg19 (chrX)	Location of SNP in hg38 (chrX)
Distal	20, 53	9.5 A/C	CACACACATATA/CTCCACA	2699555	2781514
Distal	53	9.9 G/A	CAGACAATGGCG/AATCTAT	2699968	2781927
Distal	53	10.0 C/T	TAGTCACGCC/TGATGAGCT	2700027	2781986
Distal	20	10.1 G/A	CGAGAATCCCG/AACAGCGG	2700157	2782116
Distal	20	10.2 A/G	CTCTTTCTCAA/GTCTGGTT	2700202	2782161
Distal	20	10.6 C/T	GGAGTGCTGACC/TGGTCAG	2700608	2782567
Distal	53	10.6a A/G	ACCGGTCA/GGGTTGGAAAG	2700613	2782572
Distal	20	11.0 T/C	GGTCTGACCTCT/CTTCACT	2701073	2783032
Distal	20, 53	11.1 C/T	CAAGTCCTTTC/TTCTCACT	2701185	2783144
Distal	53	12.1 C/T	AATCCCAAAC/TACCACCCA	2702143	2784102
Distal	53	12.3 C/T	CTTAAAATGC/TGTGGCTGG	2702339	2784298
Distal	53	12.6 T/C	GAACACTCAGT/CCCCTCCC	2702698	2784657
Distal	20	13.3 T/C	GATGAGCTGTT/CGGTGTAC	2703391	2785350
Distal	53	13.5 G/A	GGGAGGCAGG/ATCTGACTA	2703544	2785503
Distal	20	13.6 A/G	CAATTGAACA/GTCAGAACA	2703633	2785592
Distal	20	14.3 C/G	TCTCACTC/GTATTGCTCAG	2704335	2786294
Distal	20	14.4 T/C	CACCACACCT/CGGCTAATT	2704469	2786428
Distal	20	14.6 T/C	GTGCCTGTCCT/CCATGTTG	2704609	2786568
Distal	20	14.8 T/C	CCGGATCCAAT/CAGGACTA	2704808	2786767
Distal	53	15.0 T/C	AGCCACCCAGTT/CTATGGT	2705011	2786970
Distal	53	15.2 T/C	CTGTGATAGT/CAGCCTGAA	2705265	2787224
Proximal	53	93.1 A/G	GCTATTAAAAA/GCATTCTT	2783107	2865066
Proximal	53	93.5 T/G	CGTGTTTGT/GCCGTCCGTG	2783555	2865514
Proximal	53	94.0 A/G	GATGAATGGA/GTAAAGAAA	2784051	2866010
Proximal	53	95.4 T/G	CAAGGGAGGT/GGAAAACAG	2785428	2867387
Proximal	20, 53	96.0 G/A	GGAGGCCG/AAAGCAGGAAA	2786038	2867997
Proximal	53	97.4 A/G	GAGTCGTA/GCAACATCACT	2787485	2869444
Proximal	20, 53	97.8 C/G	CAGGCTAC/GTCTTGAATTC	2787898	2869857
Proximal	20	99.8 T/G	CAACAGCTT/GCACCATTTG	2789848	2871807
Proximal	20	100.1 T/C	CAGCCTCTT/CTCCAATGAC	2790148	2872107
Proximal	20	102.6 A/G	GTGCAGATCA/GTATCTGTG	2792662	2874621
Proximal	20	102.8 A/G	CAGCTGACA/GTCACTCAAA	2792838	2874797
