# Supplementary material for: Recombination hotspots in an extended human pseudoautosomal domain predicted from double-strand break maps and characterized by sperm-based crossover analysis
Source: PLoS Genet. 2018 Oct 8;14(10):e1007680. doi: 10.1371/journal.pgen.1007680 (PMC6193736; doi:10.1371/journal.pgen.1007680)
Supplement: S9 Table — (PDF) [file pgen.1007680.s012.pdf]

S9_Table: **Primer sequences for Ion Torrent sequencing templates**

Primer name	5' to 3' sequence	Location of 5' nt in hg19 (chrX)	Location of 5' nt in hg38 (chrX)	T_m (°C)
PAR7500F	GAAGAGGCTCCTTACTGCTC	2697499	2874797	59
X13778R	CTGGGCAAGAACACTGGTTA	2703777	2779458	
X11629F	ACACGCACATCCAGATGGAC	2701628	2785736	59
X17586R	GCCCATTGCTTTTACGGAGC	2707585	2783587	
X17401F	CAGGGAAAGGTCAACAACGC	2707422	2789544	60
X19951R	GCCTTTTGAGGAGGAAGAAC	2709953	2789381	
X19801F	ATGCTTGAGTCCCAACAGGC	2709822	2791912	64
X22701R	CCCCAACCTCAGCTCTCTCA	2712741	2791781	
X22451F3	TGTGGGCGTCCTGCAAAGAG	2712438	2794700	64
X25501R3	CTCAGAAGACCAGGCACCAG	2715545	2794397	
X25151F	GGGAAGAGGAAGCTTTGGT	2715163	2797504	51
X26301R	CAACCTACAGAGTGTTTTG	2716326	2797122	
X26243F	CTCAGATCCTCTCCGGTTTG	2716242	2798285	59
X32676R	GTGTCCCGAATAGCTCCTTA	2722675	2798201	
X28523F	TGTCCGTCATCTTCGTTACC	2718522	2804634	59
X34984R	GAAATGAAGTCCTCAGGGCA	2724983	2800481	
X34801F	CACACTGTCTGCACTGATGG	2724847	2806942	62
X37251R	GTTGCATTCCACATCCTGGC	2727281	2806806	
X36801F	CCCCAAGGCTCTTGTGAGTT	2726822	2809240	60
X39401R	CCACGGATACGATGGGAGAC	2729418	2808781	
X38251F	ATGCATGGAAAGGGTGGCTA	2728271	2811377	64
X41101R	CGTGCTTCCTTCCACCTCAG	2731130	2810230	
X40151F	TTCGTCACAGGCGAGTCAGG	2730148	2813089	64
X42851R	TTAGCACAGGTCCAGCATC	2732876	2812107	
X42651F	TTGCACAAAGAGACTGTGGC	2732677	2814835	62
X45251R	CTTTCCAAAACTGGTGCTTC	2735298	2814636	
X44151F	ACCTGGGTGACAAGGCTAAC	2734155	2817257	62
X46701R	CTGGCAGTAGGTCTCCCTAA	2736746	2816114	
X45701F	GTGTGGTGAGGACCATGGATT	2735708	2818705	64
X48501R	CCTCCCTTTGGTCTACAGCC	2738531	2817667	
X48151F	ATGGCCTCCATCTAAGCTGC	2738174	2820490	64
X50701R	GGGCTGTGTGTAAATCAGGG	2740714	2820133	
X50351aF	GTCTCCCTCCCTGTCTGACC	2740345	2822673	64
X52351R	AGACGTTGGCGAAATTGCAG	2742384	2822304	
X51983F	CACACACCTATTCCCGGC	2741982	2823941	60
X54888R	CTTGGGGACAGGTATCGGT	2744882	2826841	
X52081F	AGACCAACACACACAGGAAC	2742080	2824039	59
X58093R	ACAGCGTGGCAGTCTCGAC	2748092	2830051	
X56941F	TTCCGGACAGAGGCCAATCG	2746940	2824343	59
X62830R	AGCTGCACACATAAGGGCAT	2752829	2828899	
X62001F2	GCTCCTCATAAGCCTCAGGA	2752017	2834788	61
X64351R2	CACAGCTTCCCTCCTTTGCA	2754381	2833976	
X64301F	GTTTTTAAGGACCGAGGAGAC	2754302	2836340	59
X67251R	GCAAAATCAGATGATAGGATC	2757256	2836261	
X67151F	AGCTTATGCTATAGGTTGG	2757155	2839215	51
X70051R	TCATTAGGTAGAATGGATTC	2760081	2839114	
X69951F	CATCAGTCTTGTGCACAGAG	2759973	2842040	60
X72301R	TCTGAGGACACACTGGTTGG	2762309	2841932	
X72181F	GGAGGCATTTAATATGGTAG	2762180	2844268	57
X78205R	ACTGCTTCCTGTGTGCTCTC	2768204	2844139	
X77081F	CAAGCGTGTTATGGAGTGAT	2767080	2850163	59
X83241R	TGTGTGCAACAGAGACCCTA	2773240	2849039	
X83001F	CCAGGTGCTGTCCAATGTCG	2773030	2855199	64
X85751R	GTCGTAGAGCCTTGGCTAGC	2775804	2854989	
X85751F	TCTCGTTTCCTGCTTGCTTG	2775750	2857763	64
X88551R	AGCACACTGCAGATACTCCAC	2778580	2857709	
X88001aF	CTTCATCTCTGGTGGACGGTC	2778014	2860539	64
X90651aR	ACTCGGCCTCAGCAGACACC	2780678	2859973	
X89501F	AGCAGGTGAATTGAGGAGAC	2779505	2862637	62
X92151R	GGCTAGGAGTTTGAGCATTC	2782159	2861464	
X92028F	GAGTATCCAAGAATGCTGTG	2782027	2864118	59
X98092R	ACTAGAAGACAAAACGCTGG	2788091	2863986	
X97080F	GCTGTCCATACGGTATCTGA	2787079	2870050	59
X103595R	TGAGTGAATGTGAGGAGGTG	2793594	2869038	
X104167F	GGATAAGGAATCTGGCTGTC	2794166	2875553	57
X110158R	TTTCCCGTCTCATAAAACTG	2800157	2876125	
X109074F	CCACTGACTGTCTTCCCTAG	2799073	2882116	59
X115091R	TAAGACCTTGGAGAAATGCC	2805090	2881032	
X113156F	GCACGAAACAGGTCCTAGTG	2803155	2887049	60
X116806R	TGGCCTGAACTGTACTCTTGA	2806805	2885114	
MX115182F	AAATGAACGCTAAGCCCCAC	2805181	2888764	60
Xdup118516R	CGTCTCCAGTAACATTGCCA	2808515	2887140	
