# Supplementary material for: Recombination hotspots in an extended human pseudoautosomal domain predicted from double-strand break maps and characterized by sperm-based crossover analysis
Source: PLoS Genet. 2018 Oct 8;14(10):e1007680. doi: 10.1371/journal.pgen.1007680 (PMC6193736; doi:10.1371/journal.pgen.1007680)
Supplement: S1 Text — (PDF) [file pgen.1007680.s013.pdf]

S1_Text: **Validation of Ion Torrent data**

We validated our sequence data by comparing our genotype calls for the Belgian and French samples with those established by Mensah et al. [1] by PacBio sequencing of <5% of the total ePAR. The two data sets were entirely concordant at the fourteen sites that map uniquely to the X chromosome across all thirteen individuals. Five additional sites previously reported to map to SINEs could not be evaluated since they are not present in the hg19 X chromosome reference sequence we used to map our reads. Validation also included direct comparison of our Ion Torrent data with data from the 1000 Genomes Project [2] for the daughter (NA10847) of the ePAR carrier within CEPH pedigree 1334. We observed >99.6% concordance.

We determined haplotypes using the program PHASE [3, 4] and made use of family relationships where appropriate to determine which haplotype most likely corresponded to the ePAR. We validated this approach by comparing our ePAR data with that established by PacBio sequencing for the ~5-kb region in the previous study [1]. Haplotype calls were entirely compatible between the two data sets; however, because we were unable to include the sites that mapped to SINEs within the PacBio data, one ePAR (in Mensah's study in individuals P4 and F4, a father-son pair with the R1b ePAR) was found to be the same as that of two other ePARs (P5/F5 and P6/F6) in our data whereas it was previously distinguishable by virtue of a single variant. We observed complete concordance in ePAR assignment for the eleven cases where deduction was possible in the earlier study [1]. In the remaining two cases, one proband and his father carried the same two haplotypes, both of which were otherwise implicated as being present within ePAR from the other eleven independent father-son-(brother) groupings [1]. Our analysis suggested that this ePAR (P3/F3) is most likely to be the same as that of P5/F5 and P6/F6 over the PacBio-sequenced region (*i.e.* haplotype 2 as designated in [1]). We also noted that all five of the new cases of ePAR reported in this study matched one of the common ePAR-associated haplotypes in the previous work (*i.e.* haplotype 1). Finally, we compared our predicted and empirically derived haplotypes for each of the two sperm donors over each of the recombination assay intervals. Man 20 showed complete concordance over the ten informative sites in the distal assay region, and his haplotypes matched at six of the seven such sites in the proximal region. The corresponding data for man 53 were 6/8 and 6/7 informative sites respectively. These lower concordances might be expected as there were no first-degree relatives available to help resolve the phasing in either of these instances.

1. Mensah MA, Hestand MS, Larmuseau MH, Isrie M, Vanderheyden N, Declercq M, et al. (2014) Pseudoautosomal region 1 length polymorphism in the human population. PLoS genetics: 10. e1004578.
2. Consortium GP (2012) An integrated map of genetic variation from 1,092 human genomes. Nature 491: 56.
3. Stephens M, Smith NJ and Donnelly P (2001) A new statistical method for haplotype reconstruction from population data. Am J Hum Genet 68: 978-89.
4. Stephens M and Scheet P (2005) Accounting for decay of linkage disequilibrium in haplotype inference and missing-data imputation. Am J Hum Genet 76: 449-62.
